# Bats and their ectoparasites (Nycteribiidae and Spinturnicidae) carry diverse novel *Bartonella* genotypes, China

**DOI:** 10.1111/tbed.14357

**Published:** 2021-11-02

**Authors:** Hui‐Ju Han, Ze‐Min Li, Xia Li, Jian‐Xiao Liu, Qiu‐Ming Peng, Rui Wang, Xiao‐Lan Gu, Yuan Jiang, Chuan‐Min Zhou, Dan Li, Xiao Xiao, Xue‐Jie Yu

**Affiliations:** ^1^ State Key Laboratory of Virology School of Public Health Wuhan University Wuhan China; ^2^ Microbiological Laboratory Yantai Center for Disease Control and Prevention Yantai China; ^3^ Clinical Laboratory Xingtai Third Hospital Xingtai China; ^4^ Institute of Epidemiology Research Hubei University of Chinese Medicine Wuhan China

**Keywords:** *Bartonella*, bat, bat fly, bat mite, Nycteribiidae, Spinturnicidae

## Abstract

*Bartonella* species are facultative intracellular bacteria and recognized worldwide as emerging zoonotic pathogens. *Bartonella* were isolated or identified by polymerase chain reaction (PCR) in bats and their ectoparasites worldwide, whereas the association between them was scarce, especially in Asia. In this study, a retrospective analysis with frozen samples was carried out to identify the genetic diversity of *Bartonella* in bats and their ectoparasites and to investigate the relationships of *Bartonella* carried by bats and their ectoparasites. Bats and their ectoparasites (bat flies and bat mites) were collected from caves in Hubei Province, Central China, from May 2018 to July 2020. *Bartonella* were screened by PCR amplification and sequencing of three genes (*gltA*, *rpoB*, and *ftsZ*). Bats, bat flies, and bat mites carried diverse novel *Bartonella* genotypes with a high prevalence. The sharing of some *Bartonella* genotypes between bats and bat flies or bat mites indicated a potential role of bat flies and bat mites as vectors of bartonellae, while the higher genetic diversity of *Bartonella* in bat flies than that in bats might be due to the vertical transmission of this bacterium in bat flies. Therefore, bat flies might also act as reservoirs of *Bartonella*. In addition, human‐pathogenic *B. mayotimonesis* was identified in both bats and their ectoparasites, which expanded our knowledge on the geographic distribution of this bacterium and suggested a potential bat origin with bat flies and bat mites playing important roles in the maintenance and transmission of *Bartonella*.

## 1 INTRODUCTION

As the second largest order of mammals, bats are unique long‐lived gregarious flying mammals with a worldwide distribution, and they have been considered as the most likely sources of several viruses causing severe emerging infectious diseases in humans, including SARS‐CoV, MERS‐CoV, Nipah virus, Hendra virus, Ebola virus, Marburg virus, and the currently rampant SARS‐CoV‐2 (Han et al., 2015; Zhou et al., 2020). In the last two decades, a large number of novel viruses have been found in bats (Chen et al., 2014), whereas the study of bacterial agents in bats has been far more neglected (Mühldorfer, 2013).

*Bartonella* species are Gram‐negative, fastidious, and facultative intracellular bacteria that parasitize erythrocytes and endothelial cells of a variety of mammals, including rodents, insectivores, carnivores, ungulates, and marine mammals such as dolphins (Birtles, 2005; Dehio, 2001; Eicher & Dehio, 2012; Kosoy et al., 2010). Several *Bartonella* species are capable of causing infections in humans, including *Bartonella henselae* (cat‐scratch disease), *B. quintana* (trench fever), and *B. bacilliformis* (Carrión's disease) (Angelakis & Raoult, 2014; Jacomo et al., 2002; Rolain et al., 2004). Besides, an increasing number of novel *Bartonella* species associated with human endocarditis were reported (Edouard et al., 2015; Okaro et al., 2017).

There was evidence that bat‐borne bartonellae were associated with infections in humans and other animals. *Bartonella mayotimonensis* was first isolated from the aortic valve of a patient with endocarditis in the United States (E. Y. Lin et al., 2010). Subsequently, *Bartonella* related to *B. mayotimonensis* were identified in bats from Finland (Veikkolainen et al., 2014), the United States (Lilley et al., 2017), the United Kingdom (Concannon et al., 2005), France, and Spain (Stuckey, Boulouis, et al., 2017). In addition, some *Bartonella* genotypes found in bats from Georgia were genetically related to those identified in dogs from Thailand and humans from Poland (Urushadze et al., 2017). Moreover, a novel *Bartonella* species, *Bartonella rousetti*, was isolated from fruit bats in Nigeria, and serological studies revealed that infection with this bacterium was prevalent in the local population (Bai et al., 2018). These reports highlighted the zoonotic potential of bat‐borne bartonellae and underscored the need for expanded surveillance and investigation of these pathogens.

**FIGURE 1:**
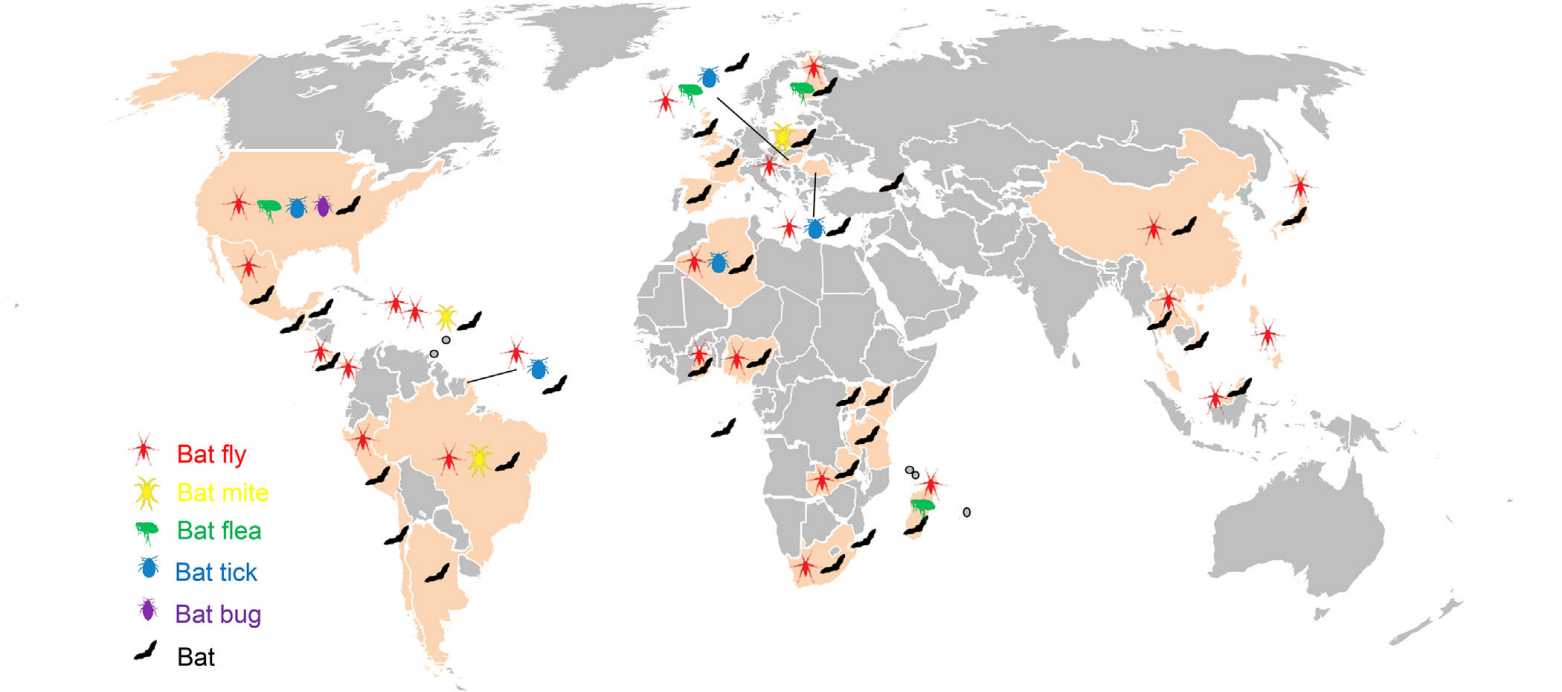
Map showing our current understanding of *Bartonella* in bats and their ectoparasites worldwide. Countries or regions with reports of *Bartonella* in bats or their ectoparasites are highlighted in gold. The map was created based on data from Table S1

Bats harbour numerous ectoparasites, including bat flies (Diptera: Nycteribiidae and Streblidae), bat bugs (Hemiptera: Cimicidae and Polyctenidae), bat fleas (Siphonaptera: Ischnopsyllidae), bat ticks (Ixodida: Ixodidae and Argasidae), and bat mites (Mesostigmata: Spinturnicidae and Macronyssidae) (Szentiványi et al., 2019). In the last decade, diverse novel *Bartonella* strains/genotypes were identified in bats and their ectoparasites worldwide (Figure 1, Table S1). Besides, a recent study inferred that bats had a great influence on both the origin and spread of *Bartonella* among other mammals and geographic regions (Mckee et al., 2021). However, there was a lack of understanding on the maintenance and transmission of *Bartonella* in bat populations. Bat flies and bat mites are obligate hematophagous ectoparasites of bats, and they may play important roles in the transmission and maintenance of bat pathogens. Bat flies were the most studied ectoparasites of bats in terms of bartonellae. The presence of identical genotypes of *Bartonella* in bat flies and their bat hosts was frequently reported (Brook et al., 2015; Dietrich et al., 2016; Judson et al., 2015; Kamani et al., 2014; Qiu et al., 2020); therefore, bat flies were generally considered as vectors for transmitting bartonellae among bats. Few studies reported the occurrence of *Bartonella* in bat mites (Hornok et al., 2012; Ikeda et al., 2020; Reeves et al., 2016), and a recent study in Poland found that identical genotypes of *Bartonella* were shared among bats and their *Spinturnix myoti* mites, indicating the possible role of bat mites in the acquisition and transmission of *Bartonella* (Szubert‐Kruszyńska et al., 2019).

Studies on the genetic diversity of *Bartonella* in bats and their ectoparasites in Asia were quite limited (Figure 1), and there was a lack of research on the relationship between them. In China, *Bartonella* were previously identified in bats from Shandong Province by our team (Han et al., 2017) and from Taiwan (J.‐W. Lin et al., 2012). However, there were hardly any reports of *Bartonella* in bat ectoparasites other than one bat fly in China (Morse et al., 2012). Considering the highly diverse novel *Bartonella* genotypes identified in bats from China in our previous work (Han et al., 2017), there was a need for further investigation on the relationship of *Bartonella* in bats and their ectoparasites.

In this study, a retrospective analysis with frozen samples was carried out to identify the genetic diversity of *Bartonella* in bats and their ectoparasites and to investigate the relationships of *Bartonella* carried by bats and their ectoparasites. Our study will provide insight into the evolution and ecology of *Bartonella* in bat populations.

## 2 MATERIALS AND METHODS

### 2.1 Ethics statement

Collection of bats for microbiological studies was approved by the Ethics Committee of the Medical School, Wuhan University (WHU2020‐YF0023), and every effort was made to minimize the discomfort of bats.

### 2.2 Sampling and species identification of bats and their ectoparasites

Frozen bats, bat flies, and bat mites stored in our laboratory, which were sampled for an ongoing programme aiming at identifying pathogens in bats, were used for the analysis of *Bartonella*. These bats were collected with mist nets, which were settled near the entrance of karst caves at sunset when bats left roosts for night feeding, and bats were collected in the next early morning. Captured bats were put in a bag, sacrificed by inhaling of ethyl ether in the field, and then transported back to the laboratory on ice as soon as possible. Once arrived at the laboratory, bats were checked for ectoparasites with forceps, with bat flies in the fur and bat mites on the membrane wings. After collection of ectoparasites, thoracic and abdominal organs of bats were collected. All the specimen were stored at −80℃ until use. Bat species were preliminary identified by morphology, and then confirmed by polymerase chain reaction (PCR) amplification and sequencing of the cytochrome B (*cytB*) gene as described previously (Li et al., 2021). Bat flies and bat mites were molecularly identified by the cytochrome oxidase subunit I (*COI*) and mitochondrial 16S rRNA genes (Bruyndonckx et al., 2009) (Table S2; Castro et al., 2002; Folmer et al., 1994).

### 2.3 Molecular detection of *Bartonella* in bats, bat flies, and bat mites

DNA was extracted from bat flies, bat mites, and bat liver tissue samples using QIAamp DNA Mini Kit (Qiagen, Valencia, CA, Spain). *Bartonella* were screened by PCR amplification and sequencing of *gltA*, *rpoB*, and *ftsZ* genes as described previously (Bai et al., 2015; Gonçalves‐Oliveira et al., 2020) (Table S2).

The PCR reaction was performed in a 50 μl mixture containing 0.25 μl 5 U/μl TaKaRa Ex Taq (TaKaRa, Shiga, Japan), 5 μl 10× Ex Taq buffer, 4 μl 25 mM MgCl_2_, 4 μl 2.5 mM dNTP mixture, 5 μl 10 μM each forward and reverse primer (Sangon Biotech, Shanghai, China), 27.75 μl nuclease‐free water, and 5 μl sample DNA. PCR was performed with one denaturation cycle at 95˚C for 5 min followed by 40 cycles at 95˚C for 30 s, 55˚C for 30 s, and 72˚C for 90 s, and an additional final cycle at 72˚C for 10 min. Each PCR assay included nuclease‐free water as a negative control. PCR products were analyzed by 1.2% agarose gel electrophoresis. Bands of expected size were excised from the gels, and purified using a Gel Extraction Kit (TSINGKE Biological Technology, Wuhan, China). The purified amplicons were cloned into the pMD19‐T vector (TaKaRa, Shiga, Japan), and at least three positive clones were selected for sequencing with the universal primers M13‐47/ M13‐48. Chromatograms were checked with Chromas 2.5.1 (Technelysium, Tewantin, QLD) to ensure sequencing accuracy. The sequences were compared with those in public databases using the Nucleotide Basic Local Alignment Search Tool (BLASTn) on the website of the National Center for Biotechnology Information (http://blast.ncbi.nlm.nih.gov/Blast.cgi).

### 2.4 Phylogenetic analysis

Sequences of interest were imported into MEGA 7.0, and primers were removed after alignment with ClustalW. Phylogenetic trees were constructed based on the neighbour‐joining method using the Kimura 2‐parameter model in MEGA 7.0, and bootstrap values were calculated using 1000 replicates.

### 2.5 Statistical analyses

Since information on the age, sex, reproduction status, or ectoparasite intensity of bats was not collected in this study, statistical analyses were not performed for these factors. Chi‐squared test and Fisher's exact test were used to evaluate the *Bartonella* prevalence by bat species, and differences were statistically significant if *p*‐values <.05.

## 3 RESULTS

### 3.1 Sampling and species identification of bats and their ectoparasites

Frozen bat tissue samples and bat ectoparasites used in this study were collected from bats sampled from three cities (Songzi, Jingmen, and Xianning) in Hubei Province, Central China, during May 2018 to July 2020. In each of the three cities, bats were sampled once from a single karst cave (Table 1).

**TABLE 1:** Sampling information and prevalence of *Bartonella* in bats, bat flies, and bat mites from Hubei Province, China

Sampling date	Sampling area	Sample type	Family	Species	Number of samples	Number of positive samples (%)	*p*‐Value
May 2018	Songzi, Hubei, China (30°17´N, 111°77´E)	Bat fly	Nycteribiidae	*Penicillidia monoceros*	15	10 (66.7)	–
				*Nycteribia* sp.	1*	1* (100)	–
		Bat	Vespertilionidae	*Myotis adversus*	4	2 (50.0)	–
				*Myotis davidii*	6	0 (0)	–
September 2019	Jingzhou, Hubei, China (30°35´N, 112°19´E)	Bat fly	Nycteribiidae	*Penicillidia monoceros*	16*	16* (100)	–
				*Nycteribia* sp.			
				*Phthiridium* sp.			
		Bat	Vespertilionidae	*Myotis davidii*	13	3 (23.1)	–
			Miniopteridae	*Miniopterus schreibersii*	3	0 (0)	–
July 2020	Xianning, Hubei, China (29°85´N, 114°30´E)	Bat fly	Nycteribiidae	*Penicillidia monoceros*	119	60 (50.4)	–
				*Nycteribia* sp.	6*	6* (100)	–
				*Phthiridium* sp.			
		Bat mite	Spinturnicidae	*Spinturnix* sp.	11*	11* (100)	–
				*Eyndhovenia* sp.			
		Bat	Vespertilionidae	*Myotis adversus*	29	1 (3.4)	<.05
				*Myotis davidii*	105	17 (16.2)	
			Rhinolophidae	*Rhinolophus pusillus*	69	3 (4.3)	
Total					397	130 (32.7)	

Note: Asterisk (*) represents pooled samples, and en‐dash (‐) indicates that no statistical analysis was performed.

**FIGURE 2:**
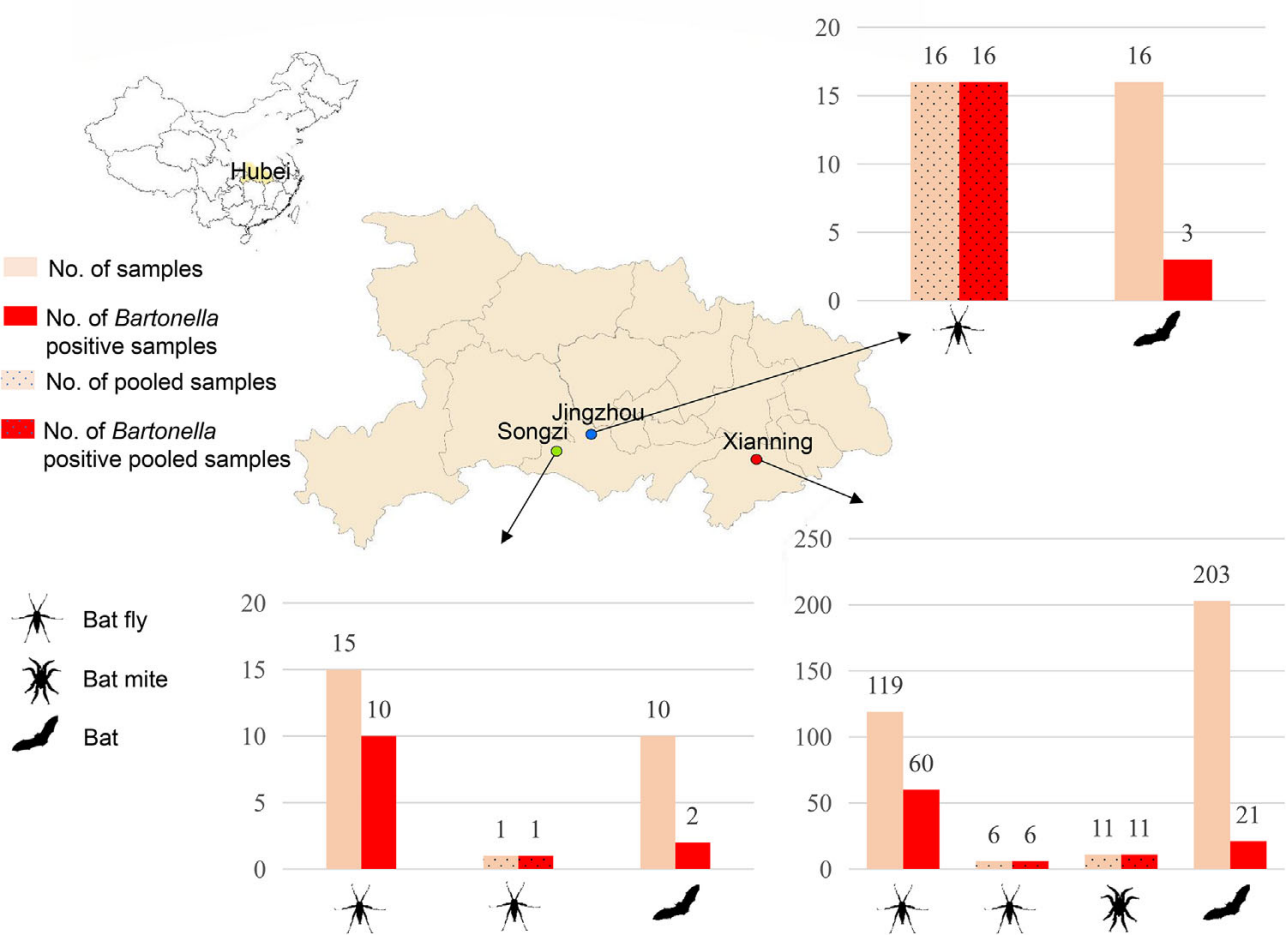
Map showing sampling of bats and their ectoparasites (bat flies and bat mites) and the positive rate of *Bartonella*

**FIGURE 3:**
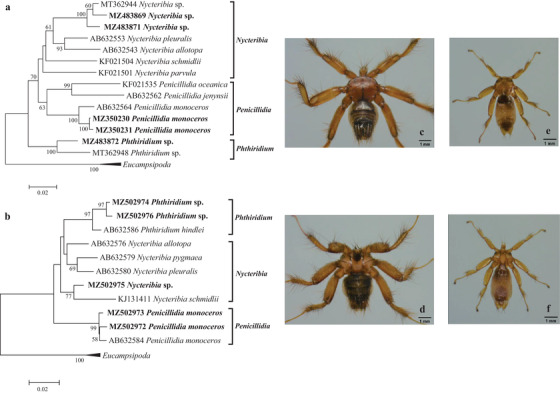
Molecular identification and photos of bat flies. Phylogenetic trees based on the (a) *COI* and (b) 16S rRNA genes of bat flies, and photos of the dorsal and ventral sides of (c and d) *Penicillidia Monoceros* and (e and f) *Nycteribia* sp. under an optical microscope

In September 2019, bat flies were collected from 16 bats sampled from a karst cave in Jingzhou, Hubei Province, China. Initially, these bat flies were divided into 16 pools (bat flies from several bats were mixed as a pool). Based on the *COI* and 16S rRNA genes, 16 bat fly pools consisted of three bat fly species belonging to three genera (*Nycteribia*, *Penicillidia*, and *Phthiridium*) in the family Nycteribiidae. Based on the *COI* gene, the three bat fly species shared 98.3%, 98.3%, and 96.4% nucleotide identity with *Penicillidia monoceros* (GenBank accession number: MW590972), *Nycteribia* sp. (Korea) (GenBank accession number: MT362944), and *Phthiridium hindlei* (GenBank accession number: AB632568), respectively. Based on the 16S rRNA gene, the three bat fly species shared 98.2%, 95.8%, and 96.3% nucleotide identity with *P*. *monoceros* (GenBank accession number: AB632585), *N*. *pleuralis* (GenBank accession number: AB632581), and *P*. *hindlei* (GenBank accession number: AB632587), respectively. Therefore, the three bat fly species found in this study were identified as *P*. *monoceros*, *Nycteribia* sp. (Korea), and a potentially novel *Phthiridium* sp. (Figures 2 and 3, Table 1). The 16 bat hosts were identified as *Myotis davidii* (13) and *Miniopterus schreibersii* (3) based on the *cytB* gene.

Due to inability to identify bat flies and bat mites using morphological keys, molecular identification was performed to confirm their species. To meet the requirement of DNA extraction, for ectoparasites collected from Songzi in 2018, and Xianning in 2020, big bat flies were individually processed, while small bat flies and bat mites were randomly pooled for *Bartonella* analysis.

In May 2018, 15 big bat flies and one small bat fly pool (about five bat flies) were collected from six bats from a karst cave in Songzi City, Hubei Province, China. The six bats were identified as *M*. *davidii* (2) and *Myotis adversus* (4) based on the *cytB* gene. Based on the *COI* and 16S rRNA genes, the 15 big bat flies were identified as *P*. *monoceros*, and the one pool of small bat flies was identified as *Nycteribia* sp. (Korea) (Figures 2 and 3, Table 1).

In July 2020, 203 bats were sampled from a karst cave in Xianning City, Hubei Province, China. Based on the *cytB* gene, the 203 bats were identified as *M*. *davidii* (105), *M*. *adversus* (29), and *Rhinolophus pusillus* (69). Bat flies collected from these bats included 119 big bat flies and six small bat fly pools (approximately 20 bat flies per pool). Based on the *COI* and 16S rRNA genes, the 119 big bat flies were all identified as *P*. *monoceros*, and the small bat fly pools consisted of *Nycteribia* sp. (Korea) and *Phthiridium* sp. (Figures 2 and 3, Table 1).

**FIGURE 4:**
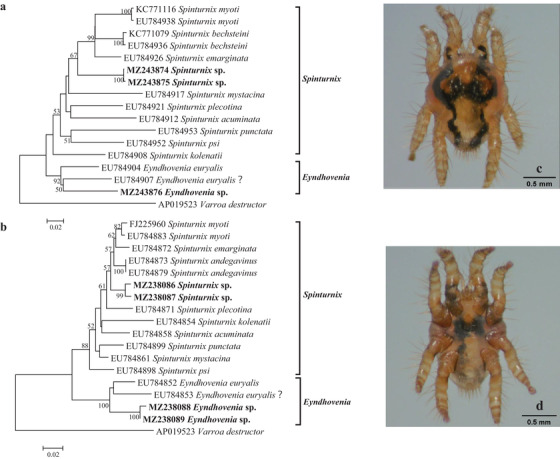
Molecular identification and photos of bat mites. Phylogenetic trees based on the (a) *COI* and (b) 16S rRNA genes of bat mites (Spinturnicidae), and photos of the dorsal and ventral sides of (c and d) *Spinturnix* sp. under an optical microscope

Bat mites collected from bats sampled in 2020 were randomly divided into 11 pools (approximately 20 bat mites per pool). Phylogenetic analysis based on the *COI* and 16S rRNA genes showed that bat mites in this study included two species belonging to the genus *Spinturnix* and the genus *Eyndhovenia* in the family Spinturnicidae, respectively. Based on the *COI* gene, *Spinturnix* sp. of this study shared 90.1% nucleotide identity with *S. myotis* (GenBank accession number: FJ225911), and *Eyndhovenia* sp. of this study shared 90.3% nucleotide identity with *Eyndhovenia euryalis* (GenBank accession number: EU784906). Based on the 16S rRNA gene, *Spinturnix* sp. of this study shared 96.7% nucleotide identity with *S*. *myotis* (GenBank accession number: FJ225969), and *Eyndhovenia* sp. of this study shared 94.3% nucleotide identity with *E. euryalis* (GenBank accession number: EU784846). Therefore, *Spinturnix* sp. and *Eyndhovenia* sp. identified in this study represented two novel species (Figures 2 and 4, Table 1).

### 3.2 Prevalence of *Bartonella* in bats, bat flies, and bat mites

*Bartonella* positive rates for bats collected in 2018, 2019, and 2020 were 20.0% (2/10), 18.8% (3/16), and 10.3% (21/203), respectively. For individual big bat flies (*P*. *monoceros*) collected in 2018 and 2020, *Bartonella* infection rates were 66.7% (10/15) and 50.4% (60/119), respectively. One small bat fly pool of 2018 (*Nycteribia* sp.), 16 bat fly pools of 2019 (*P*. *monoceros*, *Nycteribia* sp., and *Phthiridium* sp.), six small bat fly pools (*Nycteribia* sp. and *Phthiridium* sp.), and 11 bat mite pools (*Spinturnix* sp. and *Eyndhovenia* sp.) of 2020 were all positive for *Bartonella* (Figure 2, Table 1, and Table S3).

### 3.3 Molecular characterization of *Bartonella* in bats, bat flies, and bat mites

For pooled samples (bat flies and bat mites) and bats, all the samples were screened for *Bartonella* with the three genes (*gltA*, *rpoB*, and *ftsZ*). For individual big bat fly samples, they were firstly screened with the *gltA* gene. Based on the *gltA* gene, *Bartonella* identified in individual big bat flies clustered into only two groups. Therefore, representative samples were further selected for *Bartonella* characterization by the *rpoB* and *ftsZ* genes to reduce workload (Table S3).

**FIGURE 5:**
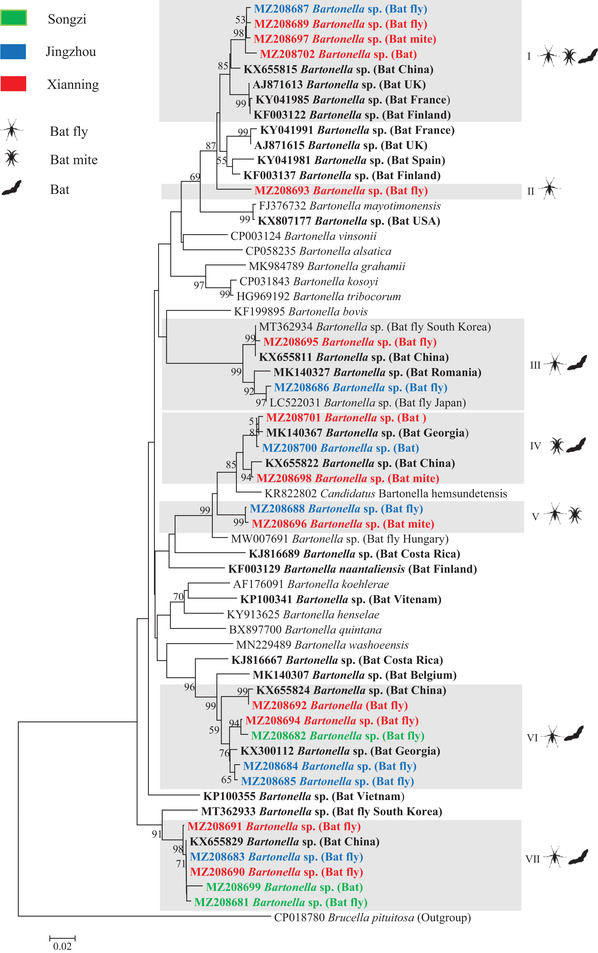
Phylogenetic analysis of bat‐borne *Bartonella* based on the *gltA* gene (327 bp). Phylogenetic tree was constructed using the neighbour‐joining method with the Kimura 2‐parameter model in MEGA 7.0. Bootstrap values more than 50% were indicated at nodes. Representative *Bartonella* sequences with hosts (bat, bat fly, and bat mite) from each sampling area (green: Songzi, Blue: Jingzhou and red: Xianning) were included in the analysis. Genotypes of *Bartonella* defined in this study are highlighted with grey box. Reference *Bartonella* sequences downloaded from GenBank were included in the analysis, and bat‐derived *Bartonella* sequences are shown in bold

**FIGURE 6:**
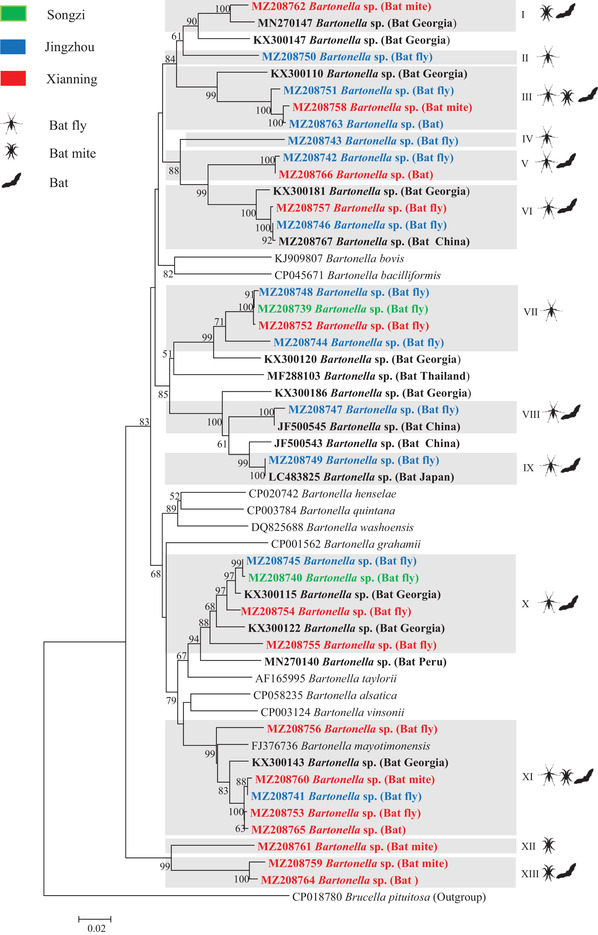
Phylogenetic analysis of bat‐borne *Bartonella* based on the *rpoB* gene (825 bp). Phylogenetic tree was constructed using the neighbour‐joining method with the Kimura 2‐parameter model in MEGA 7.0. Bootstrap values more than 50% were indicated at the nodes. Representative *Bartonella* sequences with hosts (bat, bat fly, and bat mite) from each sampling area (green: Songzi, blue: Jingzhou and red: Xianning) were included in the analysis. Genotypes of *Bartonella* defined in this study were highlighted with grey box. Reference *Bartonella* sequences downloaded from GenBank were included in the analysis, and bat‐derived *Bartonella* sequences are shown in bold

**FIGURE 7:**
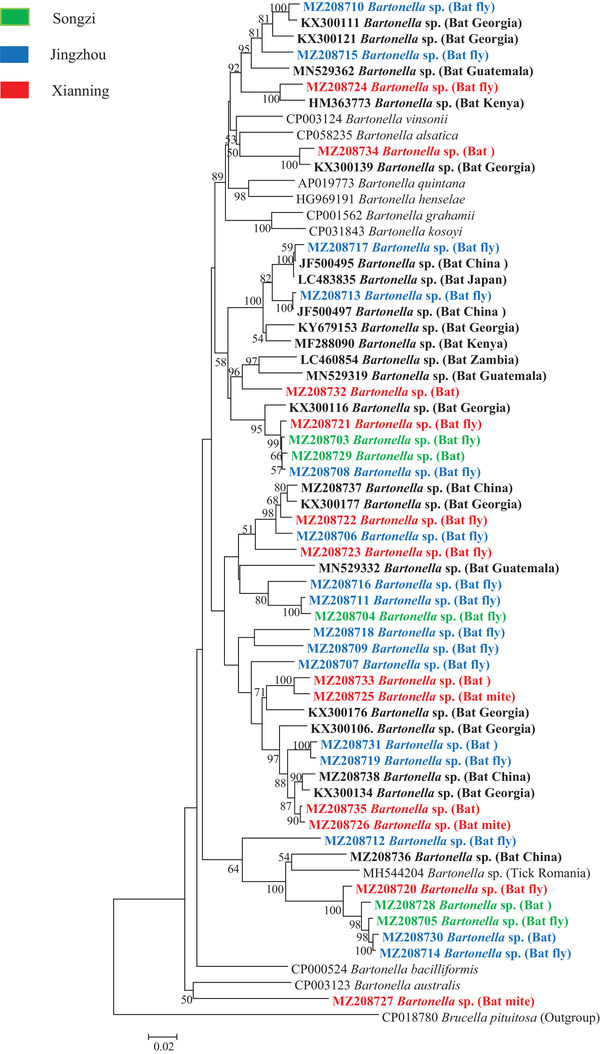
Phylogenetic analysis of bat‐borne *Bartonella* based on the *ftsZ* gene (885 bp). Phylogenetic tree was constructed using the neighbour‐joining method with the Kimura 2‐parameter model in MEGA 7.0. Bootstrap values more than 50% were indicated at the nodes. Representative *Bartonella* sequences with hosts (bat, bat fly, and bat mite) from each sampling area (green: Songzi, Blue: Jingzhou and red: Xianning) were included in the analysis. Reference *Bartonella* sequences downloaded from GenBank were included in the analysis, and bat‐derived *Bartonella* sequences are shown in bold

Phylogenetic analysis showed that bats, bat flies, and bat mites carried diverse novel genotypes of *Bartonella*, clustered with previously reported bat/bat fly‐borne bartonellae. Based on the *gltA* gene, some *Bartonella* genotypes identified in this study were closely related to those identified in bats from China, as well as in bat flies from South Korea. Based on the *rpoB* gene, some *Bartonella* genotypes identified in this study were clustered with those identified in bats from other places of China as well as from other countries such as Georgia and Japan. Based on the *ftsZ* gene, some *Bartonella* genotypes identified in this study were clustered with bat‐borne *Bartonella* from China, Georgia, Japan, and Kenya (Figures 5, 6, 7).

In 2003, La Scola et al. (2003) proposed a gene‐sequence‐based criterion for species identification of *Bartonella*: *Bartonella* isolates should be considered as a new species if the *gltA* fragment (327 bp) and the *rpoB* fragment (825 bp) share less than 96.0% and 95.4% sequence similarity, respectively, to those of validated species. According to this criterion, bartonellae identified in bats and their ectoparasites (bat flies and bat mites) in this study could be divided into seven and 13 *Bartonella* genotypes by *gltA* and *rpoB*, respectively, among which three *Bartonella* genotypes (II, V, and VII) defined by *gltA*, and 7 *Bartonella* genotypes (II, IV, V, VII, VIII, XII, and XIII) defined by *rpoB* represented novel genotypes of *Bartonella*. Based on the *gltA* gene, three, six, and three *Bartonella* genotypes were identified in bats, bat flies, and bat mites, respectively, and based on the *rpoB* gene, there were 4, 10, and 5 genotypes, respectively. Genotypes of *Bartonella* identified in bats (I, IV, and VIII by *gltA*; III, V, XI, and XIII by *rpoB*) were shared by their ectoparasites (bat flies, and bat mites). Some genotypes of *Bartonella* were identified in bat flies and bat mites, but not in bats in this study (II, III, V, and VI by *gltA*; I, II, IV, VI, VII, VIII, IX, X, and XII by *rpoB*) (Figures 5 and 6).

Based on the 825 bp *rpoB* fragment, *Bartonella* genotype (XI) identified in this study in both bats and their ectoparasites (bat flies and bat mites) shared 95.1%−95.8% nucleotide identity with *B. mayotimonesis* (GenBank accession number: FJ376736), the causative agent of human endocarditis. It was close to the cutoff value (95.4%) by the gene‐sequence‐based criteria for *Bartonella* species definition for *rpoB*; therefore, *Bartonella* genotype (XI) could be identified as *B. mayotimonesis* (Figure 6).

### 3.4 Statistical analyses

Statistical analyses of *Bartonella* prevalence by bat species were performed for bats collected from a cave in Xianning in 2020, and the *Bartonella* prevalence among the three bat species (*M. adversus*, *M. davidii*, and *R. pusillus*) was statistically significant (*χ* ^2 ^= 8.031, *p *= .018 < .05). Exactly, significant difference of the *Bartonella* prevalence was found between *M. davidii* and *R. pusillus* (*χ* ^2 ^= 5.740, *p *= .017 < .05).

### 3.5 Nucleotide sequence accession numbers

The representative sequences of this study were deposited in the GenBank database with the accession numbers: MH888178 and MW085077‐MW085079 (bat *cytB*), MZ350230‐MZ350231, MZ483869, and MZ483871‐MZ483872 (bat fly *COI*), MZ502972‐MZ502976 (bat fly 16S rRNA), MZ483874‐MZ483876 (bat mite *COI*), MZ238086‐MZ238089 (bat mite 16S rRNA), MZ208681‐MZ208702 (*Bartonella gltA*) MZ208739‐MZ208766 (*Bartonella rpoB*), and MZ208703‐MZ208735 (*Bartonella ftsZ*).

## 4 DISCUSSION

Bats, bat flies, and bat mites were collected from Hubei Province in Central China for the detection of bartonellae. The positive rate of *Bartonella* in bats in this study was 3.9% (8/203; *gltA*) and 12.8% (26/203; *gltA*, *rpoB*, and *ftsZ*). Our previous study showed that the infection rate of *Bartonella* in bat blood from Shandong Province in East China was 25.2% (27/107) by *gltA* amplification (Han et al., 2017), and previous reports of *Bartonella* prevalence in bats were 83.2% (94/113) in Peru and 45% (36/80) in Belize by *gltA* amplification (Becker et al., 2018); 33.3% (21/63) in Costa Rica (Judson et al., 2015), 33.1% (39/118) in Guatemala (Bai et al., 2011), 24.1% (27/112) in Peru (Bai et al., 2012), and 30.2% (106/331) in Kenya (Kosoy et al., 2010) by blood culture. The prevalence of *Bartonella* in bats in this study was much lower than that reported in previous studies, which may be due to the use of liver tissue rather than blood samples, as *Bartonella* generally parasitize erythrocytes and endothelial cells (Dehio, 2001), blood samples should be the first choice for PCR screening (Kosoy et al., 2018).

### 4.1 The public health significance of bat‐associated *Bartonella*

Based on the 825 bp *rpoB* fragment, the *Bartonella* genotype (XI) identified in this study in bats, bat flies, and bat mites shared 95.2%−95.7% nucleotide identify with *B. mayotimonesis* (GenBank accession number: FJ376736), which was close to the cutoff value (95.4%) of the gene sequence‐based criteria for *Bartonella* species definition proposed by La Scola et al. (2003). While based on the 327 bp *gltA* fragment, *Bartonella* genotypes identified in this study (I and II) shared 91.2%−92.6% nucleotide identify with *B. mayotimonesis* (GenBank accession number: FJ376732), which was well below the cutoff value (96.0% by *gltA*). The inconsistency of the *gltA* and *rpoB* gene might be explained by recombination events as observed in rodent‐borne bartonellae (Paziewska et al., 2011). Several studies reported the discovery of *Bartonella* species related to *B. mayotimonesis* in bats collected in the Northern hemisphere (Finland, the United States, the United Kingdom, France, and Spain) (Concannon et al., 2005; Lilley et al., 2017; Stuckey, Boulouis, et al., 2017; Veikkolainen et al., 2014). These bat‐borne bartonellae related to *B. mayotimonesis* were characterized using a short *gltA* sequence (∼327 bp). With the exception of *Bartonella* identified in bats from the United States that shared 99.7% nucleotide identity with *B. mayotimonesis* (GenBank accession number: FJ376732), *Bartonella* found in bats from European countries (Finland, the United Kingdom, Spain, and France) shared 91.2%−92.6% nucleotide identity with *B. mayotimonesis* (Table S4). In terms of phylogenetic relationship, *Bartonella* identified in bats and their ectoparasites in China were comparable to those *Bartonella* identified in bats in Europe. With the discovery of *Bartonella* that were genetically related to *B. mayotimonesis* in bats and their ectoparasites in China, our knowledge on the geographic distribution of this bacterium was expanded, and it was very likely that this bacterium was circulating in bat populations with bat flies and bat mites as vectors, even reservoirs. In addition, this study identified a number of novel *Bartonella* genotypes whose pathogenicity and public health significance were still unknown. It will be necessary to isolate these bat‐borne novel bartonellae, evaluate their pathogenicity through animal experiments, and monitor the potential spillover events through the development of detection kits.

### 4.2 Bat flies and bat mites as potential vectors and reservoirs of *Bartonella*

As described in previous reports (Judson et al., 2015; Sándor et al., 2018; Szubert‐Kruszyńska et al., 2019), some *Bartonella* genotypes were shared by bats and their ectoparasites in this study. *Bartonella* genotypes identified in bats included I, IV, and VII by *gltA*, and III, V, XI, and XIII by *rpoB*, and these genotypes were also detected in bat flies and bat mites. Although the sharing of *Bartonella* among bats and their ectoparasites could be a result of bloodsucking, more and more evidence suggested that bat flies and bat mites were probably not simple carriers of *Bartonella*. A recent study showed that the ectoparasites intensity of bats was positively correlated with *Bartonella* infection, indicating the possible role of these ectoparasites as vectors of *Bartonella* (Stuckey, Chomel, et al., 2017). In addition, previous studies reported the isolation of *Bartonella* from bat flies (Billeter et al., 2012; Nabeshima et al., 2020), confirming the viability of *Bartonella* in bat flies, and highlighting the possibility of bat flies as vectors of *Bartonella*.

The genetic diversity of bartonellae identified in bat ectoparasites was higher than that in bats in this study, which was also reported in a previous study (Judson et al., 2015). Bat fly/bat mite‐unique *Bartonella* genotypes of this study clustered with bartonellae found in bats in previous studies, it was unlikely that they were symbiotic bacteria of bat flies and bat mites. The absence of some *Bartonella* genotypes in bats might be partially explained by the low detection rate of *Bartonella* in bats in this study due to the use of liver tissue rather than blood samples for *Bartonella* detection, or due to the immune response of bats to clear certain *Bartonella* species. Moreover, bat flies have evolved a unique reproductive strategy, viviparous pupation: The larval stage develops within a female bat fly, and when internal development is complete, a larva is laid, and it immediately forms a puparium, from which an unfed adult fly emerges after a pupal stage (Figure S1) (Dick & Dittmar, 2014). During the internal developmental stages, the larva feeds on the ‘milk’ glands, which may facilitate vertical transmission of some bacteria, including *Bartonella* (Szentiványi et al., 2019). A previous study reported the identification of *Bartonella* in female bat flies and their pupae, indicating vertical transmission of *Bartonella* across developmental stages (Morse et al., 2012), and suggesting the potential roles of bat flies as reservoirs of bartonellae.

Studies on the role of bat mites in the maintenance and transmission of bartonellae were scarce, and a study in Poland reported the presence of identical *Bartonella* genotypes in bats and their mites (Szubert‐Kruszyńska et al., 2019). The role of bat flies and bat mites for *Bartonella* will be better elucidated with epidemiological investigation of *Bartonella* across all life stages of these ectoparasites and laboratory infection experiments.

Although almost all *Bartonella* genotypes identified in bats in this study were found in bat flies and bat mites, there were still some *Bartonella* genotypes unique to bats (Figure 7). The role of other ectoparasites such as hard ticks, soft ticks, fleas, and bugs in the transmission of *Bartonella* in the bat population should be investigated. Interestingly, although bartonellae are generally considered as vector‐borne bacteria, they were also identified in the saliva and faeces of bats, indicating that biting and faecal exposure might also contribute to the transmission and maintenance of bartonellae among bat population (Becker et al., 2018; Veikkolainen et al., 2014). Future studies on the infection dynamics of bartonellae will shed light on how these bacteria circulated among bats.

### 4.3 Novel bat fly and bat mite species

Bat flies are obligate bloodsucking ectoparasites that commonly parasitize in the fur and wing membranes of bats. Bat flies are divided into two families: the wingless, spider‐like Nycteribiidae, and the more traditionally fly‐like Streblidae (Dick & Patterson, 2006). Currently, the family Nycteribiidae consists of 275 species in 21 genera, and the family Streblidae consists of 227 species in 31 genera. The family Nycteribiidae has a worldwide distribution, while the family Streblidae is mainly found in Western Hemisphere (Szentiványi et al., 2019). Based on the *COI* and 16S rRNA genes, three bat fly species belonging to three genera (*Nycteribia*, *Penicillidia*, and *Phthiridium*) in the family Nycteribiidae were identified in this study, with *Phthiridium* sp. as a potentially novel bat fly species.

Mites of the family Spinturnicidae are highly specialized bat ectoparasites. The taxonomy of Spinturnicidae was mainly based on morphology, and molecular identification of this family was explored by Bruyndonckx et al. (2009), making species identification of this family possible even for non‐experts in morphology. The family Spinturnicidae includes 12 genera. The genus *Spinturnix* is the most diverse of this family and includes more than 50 named species, most of which are associated with the Old World bats (Orlova et al., 2020). Currently, only two species are recognized in the genus *Eyndhovenia*, *E. euryalis*, and *E. brachypus*, with the latter only morphologically described from *Rhinolophus rouxi* bats in China (Luong et al., 2021, Yu‐Mei Sun, 1986). Eight bat mite species of three genera (*Spinturnix*, *Eyndhovenia*, and *Paraperiglischrus*) in the family Spinturnicidae were morphologically described in China, with four species in the genus *Spinturnix* (*Spinturnix psi*, *S. myoti*, *S. sinicus*, and *S. kolenatoides*) (Rui‐Yu Ye, 1996; Tian, 2009; Yu‐Mei Sun, 1986). Bat mites of this study were molecularly identified as two species in the family Spinturnicidae, with one species belonging to the genus *Spinturnix*, and the other to the genus *Eyndhovenia*. Whether the *Spinturnix* sp. identified in this study is *S*. *sinicus* or *S. kolenatoides*, or just another novel species, and whether the *Eyndhovenia* sp. identified in this study is the *E. brachypus* or another novel species need further study. The combination of morphological characteristics and molecular data will get these bat mites better defined in the future.

Due to the lack of knowledge on the morphology of bat flies and bat mites at the beginning of the study, they were not pooled by species for *Bartonella* analysis, which left a gap in understanding the host‐vector specificity of bartonellae. However, differences in the *Bartonella* genotypes carried by different bat fly species were observed in this study. *Bartonella* genotypes (I and VII by *gltA*; VII and XI by *rpoB*) were identified in the big bat fly (*P*. *monoceros*), while for the small bat fly pools (*Nycteribia* sp., and *Phthiridium* sp.), *Bartonella* genotypes (II, III, VI, and VII by *gltA*; X and VI by *rpoB*) were identified (Table S3). Further studies will be needed to reveal the roles of each bat fly or mite species in the transmission and maintenance of *Bartonella*.

## CONFLICT OF INTEREST

The authors declare no conflict of interest.

## Supporting information

Figure S1 Bat fly (*Penicillidia monoceros*) emerging from the pupa

Table S1 Summary of current knowledge on *Bartonella* in bats and their ectoparasites worldwide

Table S2 Primers used in this study

Table S3 Detailed information on *Bartonella*‐positive samples of this study

Table S4 Estimates of evolutionary divergence between *Bartonella mayotimonensis* and closely related bat‐borne *Bartonella* species based on the *gltA* gene (327 bp)

## Data Availability

The data that support the findings of this study are openly available in GenBank at https://www.ncbi.nlm.nih.gov/genbank/

## References

[tbed14357-bib-0001] Angelakis, E. , & Raoult, D. (2014). Pathogenicity and treatment of *Bartonella* infections. International Journal of Antimicrobial Agents, 44, 16–25. 10.1016/j.ijantimicag.2014.04.006 24933445

[tbed14357-bib-0002] Bai, Y. , Hayman, D. T. S. , Mckee, C. D. , & Kosoy, M. Y. (2015). Classification of *Bartonella* strains associated with straw‐colored fruit bats (*Eidolon helvum*) across Africa using a multi‐locus sequence typing platform. PLoS Neglected Tropical Diseases, 9, e0003478. 10.1371/journal.pntd.0003478 25635826 PMC4311972

[tbed14357-bib-0003] Bai, Y. , Kosoy, M. , Recuenco, S. , Alvarez, D. , Moran, D. , Turmelle, A. , Ellison, J. , Garcia, D. L. , Estevez, A. , Lindblade, K. , & Rupprecht, C. (2011). *Bartonella* spp. in bats, guatemala. Emerging Infectious Diseases, 17, 1269–1272. 10.3201/eid1707.101867 21762584 PMC3381397

[tbed14357-bib-0004] Bai, Y. , Osinubi, M. O. V. , Osikowicz, L. , Mckee, C. , Vora, N. M. , Rizzo, M. R. , Recuenco, S. , Davis, L. , Niezgoda, M. , Ehimiyein, A. M. , Kia, G. S. N. , Oyemakinde, A. , Adeniyi, O. S. , Gbadegesin, Y. H. , Saliman, O. A. , Ogunniyi, A. , Ogunkoya, A. B. , & Kosoy, M. Y. (2018). Human exposure to novel *Bartonella* Species from contact with fruit bats. Emerging Infectious Diseases, 24, 2317–2323. 10.3201/eid2412.181204 30457529 PMC6256376

[tbed14357-bib-0005] Bai, Y. , Recuenco, S. , Gilbert, A. T. , Osikowicz, L. M. , Gómez, J. , Rupprecht, C. , & Kosoy, M. Y. (2012). Prevalence and diversity of *Bartonella* spp. in bats in Peru. American Journal of Tropical Medicine and Hygiene, 87, 518–523. 10.4269/ajtmh.2012.12-0097 22826480 PMC3435358

[tbed14357-bib-0006] Becker, D. J. , Bergner, L. M. , Bentz, A. B. , Orton, R. J. , Altizer, S. , & Streicker, D. G. (2018). Genetic diversity, infection prevalence, and possible transmission routes of *Bartonella* spp. in vampire bats. PLoS Neglected Tropical Diseases, 12, e0006786. 10.1371/journal.pntd.0006786 30260954 PMC6159870

[tbed14357-bib-0007] Billeter, S. A. , Hayman, D. T. S. , Peel, A. J. , Baker, K. , Wood, J. L. N. , Cunningham, A. , Suu‐Ire, R. , Dittmar, K. , & Kosoy, M. Y. (2012). *Bartonella* species in bat flies (Diptera: Nycteribiidae) from western Africa. Parasitology, 139, 324–329. 10.1017/S0031182011002113 22309510

[tbed14357-bib-0008] Birtles, R. J. (2005). *Bartonella*e as elegant hemotropic parasites. Annals of the New York Academy of Sciences, 1063, 270–279. 10.1196/annals.1355.044 16481527

[tbed14357-bib-0009] Brook, C. E. , Bai, Y. , Dobson, A. P. , Osikowicz, L. M. , Ranaivoson, H. C. , Zhu, Q. , Kosoy, M. Y. , & Dittmar, K. (2015). *Bartonella* spp. in fruit bats and blood‐feeding Ectoparasites in Madagascar. Plos Neglected Tropical Diseases, 9, e0003532. 10.1371/journal.pntd.0003532 25706653 PMC4337899

[tbed14357-bib-0010] Bruyndonckx, N. , Dubey, S. , Ruedi, M. , & Christe, P. (2009). Molecular cophylogenetic relationships between European bats and their ectoparasitic mites (Acari, Spinturnicidae). Molecular Phylogenetics and Evolution, 51, 227–237. 10.1016/j.ympev.2009.02.005 19236931

[tbed14357-bib-0011] Castro, L. R. , Austin, A. D. , & Dowton, M. (2002). Contrasting rates of mitochondrial molecular evolution in parasitic diptera and hymenoptera. Molecular Biology and Evolution, 19, 1100–1113. 10.1093/oxfordjournals.molbev.a004168 12082129

[tbed14357-bib-0012] Chen, L. , Liu, B. , Yang, J. , & Jin, Q.i (2014). DBatVir: The database of bat‐associated viruses. Database: The Journal of Biological Databases and Curation, 2014, bau021. 10.1093/database/bau021 24647629 PMC3958617

[tbed14357-bib-0013] Concannon, R. , Wynn‐Owen, K. , Simpson, V. R. , & Birtles, R. J. (2005). Molecular characterization of haemoparasites infecting bats (Microchiroptera) in Cornwall, UK. Parasitology, 131, 489–496. 10.1017/S0031182005008097 16174413

[tbed14357-bib-0014] Dehio, C. (2001). *Bartonella* interactions with endothelial cells and erythrocytes. Trends in Microbiology, 9, 279–285. 10.1016/S0966-842X(01)02047-9 11390243

[tbed14357-bib-0015] Dick, C. W. , & Dittmar, K. (2014). Parasitic bat flies (Diptera: Streblidae and Nycteribiidae): Host specificity and potential as vectors. In S. Klimpel & H. Mehlhorn (Eds.), Bats (Chiroptera) as vectors of diseases and parasites: Facts and myths (pp. 131–155). Springer Berlin Heidelberg.

[tbed14357-bib-0016] Dick, C. W. , & Patterson, B. D. (2006). Bat flies: Obligate ectoparasites of bats. In S. Morand , B. R. Krasnov , & R. Poulin (Eds.), Micromammals and macroparasites: From evolutionary ecology to management (pp. 179–194). Springer Japan.

[tbed14357-bib-0017] Dietrich, M. , Tjale, M. A. , Weyer, J. , Kearney, T. , Seamark, E. C. J. , Nel, L. H. , Monadjem, A. , & Markotter, W. (2016). Diversity of *Bartonella* and *Rickettsia* spp. in bats and their blood‐feeding ectoparasites from South Africa and Swaziland. Plos One, 11, e0152077. 10.1371/journal.pone.0152077 26999518 PMC4801393

[tbed14357-bib-0018] Edouard, S. , Nabet, C. , Lepidi, H. , Fournier, P.‐E. , & Raoult, D. (2015). *Bartonella*, a common cause of endocarditis: A report on 106 cases and review. Journal of Clinical Microbiology, 53, 824–829. 10.1128/JCM.02827-14 25540398 PMC4390654

[tbed14357-bib-0019] Eicher, S. C. , & Dehio, C. (2012). *Bartonella* entry mechanisms into mammalian host cells. Cellular Microbiology, 14, 1166–1173. 10.1111/j.1462-5822.2012.01806.x 22519749

[tbed14357-bib-0020] Folmer, O. , Black, M. , Hoeh, W. , Lutz, R. , & Vrijenhoek, R. (1994). DNA primers for amplification of mitochondrial cytochrome c oxidase subunit I from diverse metazoan invertebrates. Molecular Marine Biology and Biotechnology, 3, 294–299. 7881515

[tbed14357-bib-0021] Gonçalves‐Oliveira, J. , Rozental, T. , Guterres, A. , Teixeira, B. R. , Andrade‐Silva, B. E. , da Costa‐Neto, S. F. , Furtado, M. C. , Moratelli, R. , D'andrea, P. S. , & Lemos, E. R. S. (2020). Investigation of *Bartonella* spp. in Brazilian mammals with emphasis on rodents and bats from the Atlantic Forest. International Journal for Parasitology: Parasites and Wildlife, 13, 80–89. 10.1016/j.ijppaw.2020.07.004 32904298 PMC7452516

[tbed14357-bib-0022] Han, H.‐J. , Wen, H.‐L. , Zhao, L. , Liu, J.‐W. , Luo, L.‐M. , Zhou, C.‐M. , Qin, X.‐R. , Zhu, Y.‐L. , Zheng, X.‐X. , & Yu, X.‐J. (2017). Novel *Bartonella* species in insectivorous bats, northern China. Plos One, 12, e0167915. 10.1371/journal.pone.0167915 28081122 PMC5231389

[tbed14357-bib-0023] Han, H.‐J. , Wen, H.‐L. , Zhou, C.‐M. , Chen, F.‐F. , Luo, L.i‐M. , Liu, J.‐W. , & Yu, X.‐J. (2015). Bats as reservoirs of severe emerging infectious diseases. Virus Research, 205, 1–6. 10.1016/j.virusres.2015.05.006 25997928 PMC7132474

[tbed14357-bib-0024] Hornok, S. , Kovács, R. , Meli, M. L. , Gönczi, E. , Hofmann‐Lehmann, R. , Kontschán, J. , Gyuranecz, M. , Dán, Á. , & Molnár, V. (2012). First detection of *Bartonellae* in a broad range of bat ectoparasites. Veterinary Microbiology, 159, 541–543. 10.1016/j.vetmic.2012.04.003 22551590

[tbed14357-bib-0025] Ikeda, P. , Marinho Torres, J. , Perles, L. , Lourenço, E. C. , Herrera, H. M. , De Oliveira, C. E. , Zacarias Machado, R. , & André, M. R. (2020). Intra‐ and inter‐host assessment of *Bartonella* diversity with focus on non‐hematophagous bats and associated ectoparasites from Brazil. Microorganisms, 8, 1822. 10.3390/microorganisms8111822 PMC7699196 33227996

[tbed14357-bib-0026] Jacomo, V. , Kelly, P. J. , & Raoult, D. (2002). Natural history of *Bartonella* infections (an exception to Koch's postulate). Clinical and Diagnostic Laboratory Immunology, 9, 8–18. 11777823 10.1128/CDLI.9.1.8-18.2002 PMC119901

[tbed14357-bib-0027] Judson, S. D. , Frank, H. K. , & Hadly, E. A. (2015). *Bartonella*e are prevalent and diverse in costa rican bats and bat Flies. Zoonoses and Public Health, 62, 609–617. 10.1111/zph.12188 25810119

[tbed14357-bib-0028] Kamani, J. , Baneth, G. , Mitchell, M. , Mumcuoglu, K. Y. , Gutiérrez, R. , & Harrus, S. (2014). *Bartonella* species in bats (Chiroptera) and bat flies (Nycteribiidae) from Nigeria, West Africa. Vector Borne and Zoonotic Diseases, 14, 625–632. 10.1089/vbz.2013.1541 25229701 PMC4170809

[tbed14357-bib-0029] Kosoy, M. , Bai, Y. , Lynch, T. , Kuzmin, I. V. , Niezgoda, M. , Franka, R. , Agwanda, B. , Breiman, R. F. , & Rupprecht, C. E. (2010). *Bartonella* spp. in bats, Kenya. Emerging Infectious Diseases, 16, 1875–1881. 10.3201/eid1612.100601 21122216 PMC3294596

[tbed14357-bib-0030] Kosoy, M. , Mckee, C. , Albayrak, L. , & Fofanov, Y. (2018). Genotyping of *Bartonella* bacteria and their animal hosts: Current status and perspectives. Parasitology, 145, 543–562. 10.1017/S0031182017001263 28764816

[tbed14357-bib-0031] La Scola, B. , Zeaiter, Z. , Khamis, A. , & Raoult, D. (2003). Gene‐sequence‐based criteria for species definition in bacteriology: The *Bartonella* paradigm. Trends in Microbiology, 11, 318–321. 10.1016/S0966-842X(03)00143-4 12875815

[tbed14357-bib-0032] Li, Z.‐M. , Xiao, X. , Zhou, C.‐M. , Liu, J.‐X. , Gu, X.‐L. , Fang, L.‐Z. , Liu, B.‐Y. , Wang, L.‐R. , Yu, X.‐J. , & Han, H.‐J. (2021). Human‐pathogenic relapsing fever *Borrelia* found in bats from Central China phylogenetically clustered together with relapsing fever borreliae reported in the New World. PLoS Neglected Tropical Diseases, 15, e0009113. 10.1371/journal.pntd.0009113 33735240 PMC7971464

[tbed14357-bib-0033] Lilley, T. M. , Wilson, C. A. , Bernard, R. F. , Willcox, E. V. , Vesterinen, E. J. , Webber, Q. M. R. , Kurpiers, L. , Prokkola, J. M. , Ejotre, I. , Kurta, A. , Field, K. A. , Reeder, D. M. , & Pulliainen, A. T. (2017). Molecular detection of *Candidatus Bartonella* mayotimonensis in North American bats. Vector Borne and Zoonotic Diseases, 17, 243–246. 10.1089/vbz.2016.2080 28165925

[tbed14357-bib-0034] Lin, J.‐W. , Hsu, Y.‐M. , Chomel, B. B. , Lin, L.‐K. , Pei, J.‐C. , Wu, S.‐H. , & Chang, C.‐C. (2012). Identification of novel *Bartonella* spp. in bats and evidence of Asian gray shrew as a new potential reservoir of *Bartonella* . Veterinary Microbiology, 156, 119–126. 10.1016/j.vetmic.2011.09.031 22005177 PMC7126237

[tbed14357-bib-0035] Lin, E. Y. , Tsigrelis, C. , Baddour, L. M. , Lepidi, H. , Rolain, J.‐M. , Patel, R. , & Raoult, D. (2010). *Candidatus Bartonella* mayotimonensis and endocarditis. Emerging Infectious Diseases, 16, 500–503. 10.3201/eid1603.081673 20202430 PMC3321999

[tbed14357-bib-0036] Luong, N. T. , Orlova, M. V. , Manh, V.u Q. , Loan, H.o T. , & Thong, V. D. (2021). First record of eyndhovenia (Mesostigmata: Gamasina: Spinturnicidae) from Vietnam. Parasitology International, 82, 102301. 10.1016/j.parint.2021.102301 33607283

[tbed14357-bib-0037] Mckee, C. D. , Bai, Y. , Webb, C. T. , & Kosoy, M. Y. (2021). Bats are key hosts in the radiation of mammal‐associated *Bartonella* bacteria. Infection, Genetics and Evolution, 89, 104719. 10.1016/j.meegid.2021.104719 PMC10915969 33444855

[tbed14357-bib-0038] Morse, S. F. , Olival, K. J. , Kosoy, M. , Billeter, S. , Patterson, B. D. , Dick, C. W. , & Dittmar, K. (2012). Global distribution and genetic diversity of *Bartonella* in bat flies (Hippoboscoidea, Streblidae, Nycteribiidae). Infection, Genetics and Evolution, 12, 1717–1723. 10.1016/j.meegid.2012.06.009 22771358

[tbed14357-bib-0039] Mühldorfer, K. (2013). Bats and bacterial pathogens: A review. Zoonoses and Public Health, 60, 93–103. 10.1111/j.1863-2378.2012.01536.x 22862791

[tbed14357-bib-0040] Nabeshima, K. , Sato, S. , Kabeya, H. , Komine, N. , Nanashima, R. , Takano, A. , Shimoda, H. , Maeda, K. , Suzuki, K. , & Maruyama, S. (2020). Detection and phylogenetic analysis of *Bartonella* species from bat flies on eastern bent‐wing bats (*Miniopterus fuliginosus*) in Japan. Comparative Immunology, Microbiology and Infectious Diseases, 73, 101570. 10.1016/j.cimid.2020.101570 33129175

[tbed14357-bib-0041] Okaro, U. , Addisu, A. , Casanas, B. , & Anderson, B. (2017). *Bartonella* Species, an emerging cause of blood‐culture‐negative endocarditis. Clinical Microbiology Reviews, 30, 709–746. 10.1128/CMR.00013-17 28490579 PMC5475225

[tbed14357-bib-0042] Orlova, M. V. , Laverty, T. M. , Reeves, W. K. , Gratton, E. M. , & Davies, M. L. (2020). The first record of the spinturnicid mite *Spinturnix kolenatii* Oudemans, 1910 (Mesostigmata: Gamasina: Spinturnicidae) from the long‐tailed serotine bat *Eptesicus hottentotus* A. Smith, 1833 (Chiroptera: Vespertilionidae) in Africa. International Journal of Acarology, 46, 160–164. 10.1080/01647954.2020.1731596

[tbed14357-bib-0043] Paziewska, A. , Harris, P. D. , Zwolińska, L. , Bajer, A. , & Siński, E. (2011). Recombination within and between species of the alpha proteobacterium *Bartonella* infecting rodents. Microbial Ecology, 61, 134–145. 10.1007/s00248-010-9735-1 20740281 PMC3011088

[tbed14357-bib-0044] Qiu, Y. , Kajihara, M. , Nakao, R. , Mulenga, E. , Harima, H. , Hang'ombe, B. M. , Eto, Y. , Changula, K. , Mwizabi, D. , Sawa, H. , Higashi, H. , Mweene, A. , Takada, A. , Simuunza, M. , & Sugimoto, C. (2020). Isolation of *Candidatus* Bartonella rousetti and Other Bat‐associated *Bartonella*e from Bats and Their Flies in Zambia. Pathogens, 9, 469. 10.3390/pathogens9060469 PMC7350321 32545824

[tbed14357-bib-0045] Reeves, W. K. , Beck, J. , Orlova, M. V. , Daly, J. L. , Pippin, K. , Revan, F. , & Loftis, A. D. (2016). Ecology of bats, their ectoparasites, and associated pathogens on Saint Kitts Island. Journal of Medical Entomology, 53, 1218–1225. 10.1093/jme/tjw078 27282816

[tbed14357-bib-0046] Rolain, J. M. , Brouqui, P. , Koehler, J. E. , Maguina, C. , Dolan, M. J. , & Raoult, D. (2004). Recommendations for treatment of human infections caused by *Bartonella* species. Antimicrobial Agents and Chemotherapy, 48, 1921–1933. 10.1128/AAC.48.6.1921-1933.2004 15155180 PMC415619

[tbed14357-bib-0047] Rui‐Yu Ye, L.‐M. M. (1996). A new species of *Spinturnix* and a new record of *Steatonyssus* from China (Acari: Spinturnicidae, Macronyssidae). Acta Zootaxonomica Sinica, 21, 421–424.

[tbed14357-bib-0048] Sándor, A. D. , Földvári, M. , Krawczyk, A. I. , Sprong, H. , Corduneanu, A. , Barti, L. , Görföl, T. , Estók, P. , Kováts, D. , Szekeres, S. , László, Z. , Hornok, S. , & Földvári, G. (2018). Eco‐epidemiology of novel *Bartonella* genotypes from parasitic flies of insectivorous bats. Microbial Ecology, 76, 1076–1088. 10.1007/s00248-018-1195-z 29705820

[tbed14357-bib-0049] Stuckey, M. J. , Boulouis, H.‐J. , Cliquet, F. , Picard‐Meyer, E. , Servat, A. , Aréchiga‐Ceballos, N. , Echevarría, J. E. , & Chomel, B. B. (2017). Potentially zoonotic *Bartonella* in bats from France and Spain. Emerging Infectious Diseases, 23, 539–541. 10.3201/eid2303.160934 28221109 PMC5382759

[tbed14357-bib-0050] Stuckey, M. J. , Chomel, B. B. , Galvez‐Romero, G. , Olave‐Leyva, J. I. , Obregón‐Morales, C. , Moreno‐Sandoval, H. , Aréchiga‐Ceballos, N. , Salas‐Rojas, M. , & Aguilar‐Setién, A. (2017). *Bartonella* infection in hematophagous, insectivorous, and phytophagous bat populations of Central Mexico and the Yucatan Peninsula. American Journal of Tropical Medicine and Hygiene, 97, 413–422. 10.4269/ajtmh.16-0680 28722567 PMC5544074

[tbed14357-bib-0051] Szentiványi, T. , Christe, P. , & Glaizot, O. (2019). Bat Flies and their microparasites: Current knowledge and distribution. Frontiers in Veterinary Science, 6, 115. 10.3389/fvets.2019.00115 31106212 PMC6492627

[tbed14357-bib-0052] Szubert‐Kruszyńska, A. , Stańczak, J. , Cieniuch, S. , Podsiadły, E. , Postawa, T. , & Michalik, J. (2019). *Bartonella* and *Rickettsia* infections in haematophagous *Spinturnix myoti* mites (Acari: Mesostigmata) and their bat host, *Myotis myotis* (Yangochiroptera: Vespertilionidae), from Poland. Microbial Ecology, 77, 759–768. 10.1007/s00248-018-1246-5 30151669 PMC6469609

[tbed14357-bib-0053] Tian, Z. (2009). Taxonomic Studies on Ectoparasites Gamasid of Bats in China (Acari: Mesostigmata, Gamasina) Guizhou University.

[tbed14357-bib-0054] Urushadze, L. , Bai, Y. , Osikowicz, L. , Mckee, C. , Sidamonidze, K. , Putkaradze, D. , Imnadze, P. , Kandaurov, A. , Kuzmin, I. , & Kosoy, M. (2017). Prevalence, diversity, and host associations of *Bartonella* strains in bats from Georgia (Caucasus). PLoS Neglected Tropical Diseases, 11, e0005428. 10.1371/journal.pntd.0005428 28399125 PMC5400274

[tbed14357-bib-0055] Veikkolainen, V. , Vesterinen, E. J. , Lilley, T. M. , & Pulliainen, A. T. (2014). Bats as reservoir hosts of human bacterial pathogen, *Bartonella mayotimonensis* . Emerging Infectious Diseases, 20, 960–967. 10.3201/eid2006.130956 24856523 PMC4036794

[tbed14357-bib-0056] Yu‐Mei Sun, L.‐L. W. , & Wang, D.‐Q. (1986). A new species of *Eyndhovenia* from Fujian (Mesostigmata: Spinturnicidae). Acta Zootaxonomica Sinica, 11, 194–197.

[tbed14357-bib-0057] Zhou, P. , Yang, X.‐L. , Wang, X.‐G. , Hu, B. , Zhang, L. , Zhang, W. , Si, H.‐R. , Zhu, Y. , Li, B. , Huang, C.‐L. , Chen, H.‐D. , Chen, J. , Luo, Y. , Guo, H. , Jiang, R.‐D.i , Liu, M.‐Q. , Chen, Y. , Shen, X.u‐R. , Wang, X. … Shi, Z.‐L. (2020). A pneumonia outbreak associated with a new coronavirus of probable bat origin. Nature, 579, 270–273. 10.1038/s41586-020-2012-7 32015507 PMC7095418

