# Supplementary figures and images for: Bats and their ectoparasites (Nycteribiidae and Spinturnicidae) carry diverse novel Bartonella genotypes, China
Source: Transbound Emerg Dis. 2021 Nov 2;69(4):e845–58. doi: 10.1111/tbed.14357 (PMC9543326; doi:10.1111/tbed.14357)

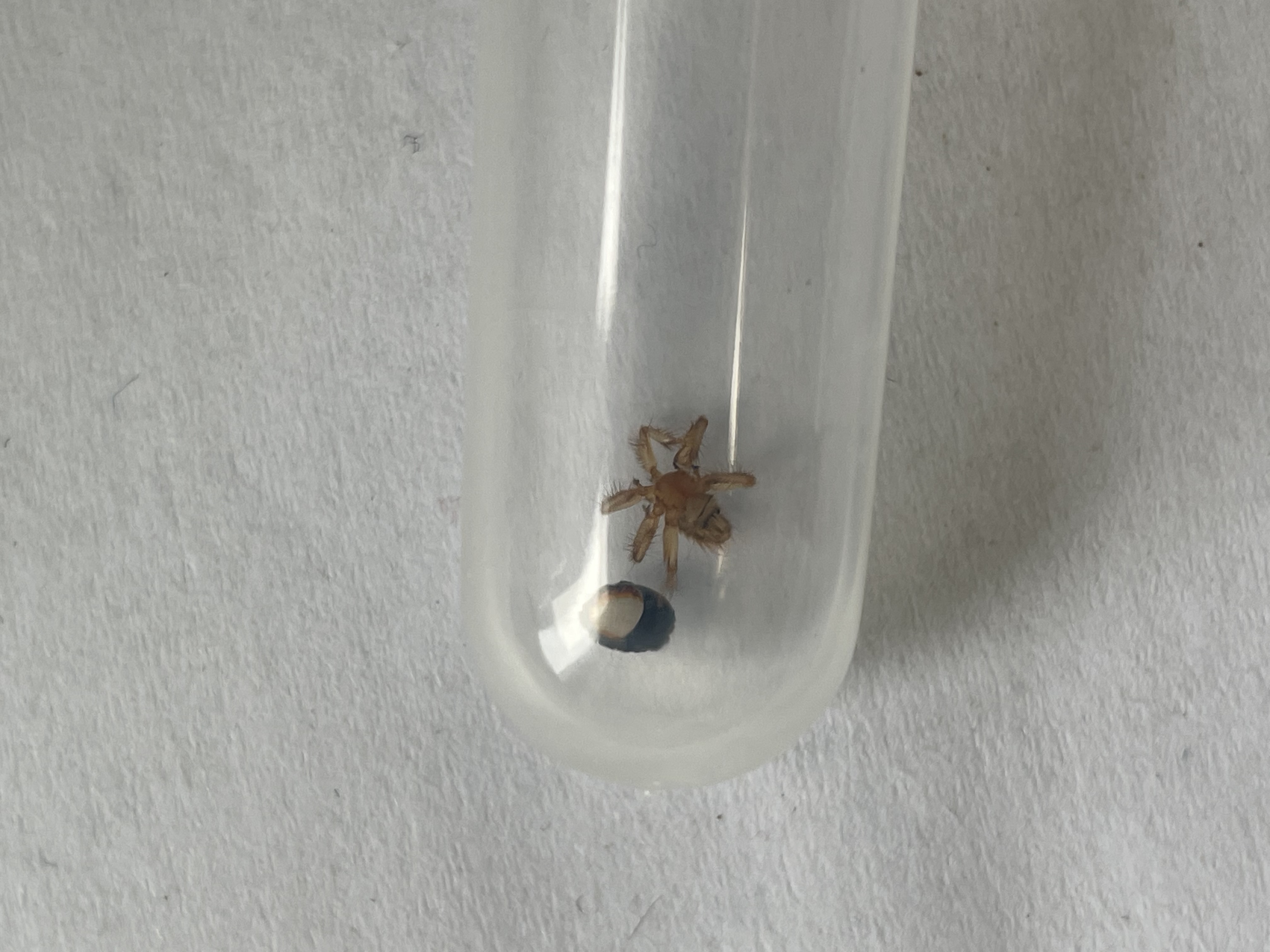

Supplement: Supplementary file 1 — Figure S1 Bat fly (Penicillidia monoceros) emerging from the pupa [file TBED-69-e845-s002.jpg]
