# Supplementary material for: Bats and their ectoparasites (Nycteribiidae and Spinturnicidae) carry diverse novel Bartonella genotypes, China
Source: Transbound Emerg Dis. 2021 Nov 2;69(4):e845–58. doi: 10.1111/tbed.14357 (PMC9543326; doi:10.1111/tbed.14357)
Supplement: Supplementary file 2 — Table S1 Summary of current knowledge on Bartonella in bats and their ectoparasites worldwide [file TBED-69-e845-s004.docx]

Table S1. Summary of current knowledge on *Bartonella* in bats and their ectoparasites worldwide.

| **Sample type** | **Sample species** | **Sample family** | **Country** | **Continent** | **Literature** |
| --- | --- | --- | --- | --- | --- |
| Bat | ND | ND | Algeria | Africa | Leulmi, Hamza et al. 2016 |
| Bat fly | *Nycteribiidae* spp. | Nycteribiidae | Algeria | Africa | Leulmi, Hamza et al. 2016 |
| Bat tick | *Ixodes vespertilionis* | Ixodidae | Algeria | Africa | Leulmi, Hamza et al. 2016 |
| Bat | *Eidolon helvum* | Pteropodidae | Annobón | Africa | Bai, Ying et al. 2015 |
| Bat | *Eidolon helvum* | Pteropodidae | Ghana | Africa | Bai, Ying et al. 2015 |
| Bat fly | *Cyclopodia greeffi* | Nycteribiidae | Ghana | Africa | Billeter, Sarah A et al. 2012 |
| Bat | *Eidolon helvum* | Pteropodidae | Kenya | Africa | Bai, Ying et al. 2015 |
| Bat | *Coleura afra* | Emballonuridae | Kenya | Africa | Kosoy, Michael et al. 2010 |
| Bat | *Eidolon helvum* | Pteropodidae | Kenya | Africa | Kosoy, Michael et al. 2010 |
| Bat | *Hipposideros commersoni* | Hipposideridae | Kenya | Africa | Kosoy, Michael et al. 2010 |
| Bat | *Miniopterus* spp. | Miniopteridae | Kenya | Africa | Kosoy, Michael et al. 2010 |
| Bat | *Rousettus aegyptiacus* | Pteropodidae | Kenya | Africa | Kosoy, Michael et al. 2010 |
| Bat | *Triaenops persicus* | Hipposideridae | Kenya | Africa | Kosoy, Michael et al. 2010 |
| Bat | *Eidolon dupreanum* | Pteropodidae | Madagascar | Africa | Brook, Cara E et al. 2015 |
| Bat flea | *Thaumapsylla* sp. | Ischnopsyllidae | Madagascar | Africa | Brook, Cara E et al. 2015 |
| Bat fly | *Cyclopodia dubia* | Nycteribiidae | Madagascar | Africa | Brook, Cara E et al. 2015 |
| Bat fly | *Basilia (Paracyclopodia )* sp. | Nycteribiidae | Madagascar | Africa | Wilkonson, David A et al. 2016 |
| Bat fly | *Cyclopodia dubia* | Nycteribiidae | Madagascar | Africa | Wilkonson, David A et al. 2016 |
| Bat fly | *Eucampsipoda theodori* | Nycteribiidae | Madagascar | Africa | Wilkonson, David A et al. 2016 |
| Bat fly | *Nycteribia stylidiopsis* | Nycteribiidae | Madagascar | Africa | Wilkonson, David A et al. 2016 |
| Bat fly | *Penicillidia leptothrinax* | Nycteribiidae | Madagascar | Africa | Wilkonson, David A et al. 2016 |
| Bat fly | *Penicillidia* sp. | Nycteribiidae | Madagascar | Africa | Wilkonson, David A et al. 2016 |
| Bat | *Eidolon helvum* | Pteropodidae | Nigeria | Africa | Bai, Ying et al. 2015 |
| Bat | *Rousettus aegyptiacus* | Pteropodidae | Nigeria | Africa | Bai, Ying et al. 2018 |
| Bat | *Chaerephon nigeriae* | Molossidae | Nigeria | Africa | Kamani, Joshua et al 2014 |
| Bat | *Eidolon helvum* | Pteropodidae | Nigeria | Africa | Kamani, Joshua et al 2014 |
| Bat | *Epomorphorus* spp. | [Pteropodidae](https://animaldiversity.org/accounts/Pteropodidae/) | Nigeria | Africa | Kamani, Joshua et al 2014 |
| Bat | *Micropterus* spp. | Phyllostomidae | Nigeria | Africa | Kamani, Joshua et al 2014 |
| Bat | *Rhinolophus* spp. | Rhinolophidae | Nigeria | Africa | Kamani, Joshua et al 2014 |
| Bat fly | *Eucampsipoda africana* | Nycteribiidae | Nigeria | Africa | Bai, Ying et al. 2018 |
| Bat fly | *Cyclopodia greeffi* | Nycteribiidae | Nigeria | Africa | Kamani, Joshua et al 2014 |
| Bat | *Epomophorus wahlbergi* | Pteropodidae | South Africa | Africa | Dietrich, Muriel et al. 2016 |
| Bat | *Miniopterus natalensis* | Miniopteridae | South Africa | Africa | Dietrich, Muriel et al. 2016 |
| Bat | *Rousettus aegyptiacus* | Pteropodidae | South Africa | Africa | Dietrich, Muriel et al. 2016 |
| Bat fly | *Eucampsipoda* spp. | Nycteribiidae | South Africa | Africa | Dietrich, Muriel et al. 2016 |
| Bat | *Nycteris thebaica* | Nycteridae | Swaziland | Africa | Dietrich, Muriel et al. 2016 |
| Bat | *Eidolon helvum* | Pteropodidae | Tanzania | Africa | Bai, Ying et al. 2015 |
| Bat | *Eidolon helvum* | Pteropodidae | Uganda | Africa | Bai, Ying et al. 2015 |
| Bat | *Macronycteris vittatus* | Hipposideridae | Zambia | Africa | Qiu, Yongjin et al. 2020 |
| Bat | *Rousettus aegyptiacus* | Pteropodidae | Zambia | Africa | Qiu, Yongjin et al. 2020 |
| Bat fly | *Eucampsipoda africana* | Hipposideridae | Zambia | Africa | Qiu, Yongjin et al. 2020 |
| Bat | *Myotis fimbriatus* | Vespertilionidae | China | Asia | Han, Hui-Ju et al. 2017 |
| Bat | *Myotis pequinius* | Vespertilionidae | China | Asia | Han, Hui-Ju et al. 2017 |
| Bat | *Myotis ricketti* | Vespertilionidae | China | Asia | Han, Hui-Ju et al. 2017 |
| Bat | *Rhinolophus ferrumequinum* | Rhinolophidae | China | Asia | Han, Hui-Ju et al. 2017 |
| Bat | *Rhinolophus pusillus* | Rhinolophidae | China | Asia | Han, Hui-Ju et al. 2017 |
| Bat | *Miniopterus schreibersii* | Miniopteridae | China | Asia | Lin, Jen-Wei et al. 2012 |
| Bat fly | *Eucampsipoda latisterna* | Nycteribiidae | China | Asia | Morse, Solon F et al 2012 |
| Bat | *Eptesicus serotinus* | Vespertilionidae | Georgia | Asia | Bai, Ying et al. 2017 |
| Bat | *Miniopterus schreibersii* | Miniopteridae | Georgia | Asia | Bai, Ying et al. 2017 |
| Bat | *Myotis blythii* | Vespertilionidae | Georgia | Asia | Bai, Ying et al. 2017 |
| Bat | *Myotis emarginatus* | Vespertilionidae | Georgia | Asia | Bai, Ying et al. 2017 |
| Bat | *Pipistrellus pygmaeus* | Vespertilionidae | Georgia | Asia | Bai, Ying et al. 2017 |
| Bat | *Rhinolophus euryale* | Rhinolophidae | Georgia | Asia | Bai, Ying et al. 2017 |
| Bat | *Rhinolophus ferrimequinum* | Rhinolophidae | Georgia | Asia | Bai, Ying et al. 2017 |
| Bat | *Eptesicus serotinus* | Vespertilionidae | Georgia | Asia | Urushadze, Lela et al. 2017 |
| Bat | *Miniopterus schreibersii* | Miniopteridae | Georgia | Asia | Urushadze, Lela et al. 2017 |
| Bat | *Myotis blythii* | Vespertilionidae | Georgia | Asia | Urushadze, Lela et al. 2017 |
| Bat | *Myotis emarginatus* | Vespertilionidae | Georgia | Asia | Urushadze, Lela et al. 2017 |
| Bat | *Pipistrellus pygmaeus* | Vespertilionidae | Georgia | Asia | Urushadze, Lela et al. 2017 |
| Bat | *Rhinolophus euryale* | Rhinolophidae | Georgia | Asia | Urushadze, Lela et al. 2017 |
| Bat | *Rhinolophus ferrumequinum* | Rhinolophidae | Georgia | Asia | Urushadze, Lela et al. 2017 |
| Bat | *Miniopterus fuliginosus* | Miniopteridae | Japan | Asia | Nabeshima, Kei et al. 2020 |
| Bat fly | *Nycteribia allotopa* | Nycteribiidae | Japan | Asia | Nabeshima, Kei et al. 2020 |
| Bat fly | *Nycteribia* sp. | Nycteribiidae | Japan | Asia | Nabeshima, Kei et al. 2020 |
| Bat fly | *Penicilidia jenynsii* | Nycteribiidae | Japan | Asia | Nabeshima, Kei et al. 2020 |
| Bat fly | *Phthiridium (Stylidia)* sp. | Nycteribiidae | Laos | Asia | Morse, Solon F et al. 2012 |
| Bat | *Pteropushypomelanus* | Mormoopidae | Malaysia | Asia | Hou, S L et al. 2018 |
| Bat fly | *Basilia (Tripselia) coronata* | Nycteribiidae | Malaysia | Asia | Morse, Solon F et al. 2012 |
| Bat fly | *Cyclopodia horsfieldii* | Nycteribiidae | Malaysia | Asia | Morse, Solon F et al. 2012 |
| Bat fly | *Phthiridium (Stylidia) fraterna* | Nycteribiidae | Malaysia | Asia | Morse, Solon F et al. 2012 |
| Bat fly | *Cyclopodia simulans* | Nycteribiidae | Philippines | Asia | Morse, Solon F et al. 2012 |
| Bat fly | *Leptocyclopodia* sp. | Nycteribiidae | Philippines | Asia | Morse, Solon F et al. 2012 |
| Bat | *Chaerephon plicatus* | Molossidae | Thailand | Asia | McKee, Clifton D et al. 2017 |
| Bat | *Hipposideros armiger* | Hipposideridae | Thailand | Asia | McKee, Clifton D et al. 2017 |
| Bat | *Hipposideros fulvus* | Hipposideridae | Thailand | Asia | McKee, Clifton D et al. 2017 |
| Bat | *Hipposideros larvatus* | Hipposideridae | Thailand | Asia | McKee, Clifton D et al. 2017 |
| Bat | *Taphozous melanopogon* | Emballonuridae | Thailand | Asia | McKee, Clifton D et al. 2017 |
| Bat | *Hipposideros armiger* | Hipposideridae | Vietnam | Asia | Anh, Pham H et al. 2015 |
| Bat | *Hipposideros larvatui* | Hipposideridae | Vietnam | Asia | Anh, Pham H et al. 2015 |
| Bat | *Megaderma lyra* | Pteropodidae | Vietnam | Asia | Anh, Pham H et al. 2015 |
| Bat | *Megaderma spasma* | Pteropodidae | Vietnam | Asia | Anh, Pham H et al. 2015 |
| Bat | *Megaerops niphanae* | Pteropodidae | Vietnam | Asia | Anh, Pham H et al. 2015 |
| Bat | *Rhinolophus acuminatus* | Rhinolophidae | Vietnam | Asia | Anh, Pham H et al. 2015 |
| Bat | *Rhinolophus chaseli* | Rhinolophidae | Vietnam | Asia | Anh, Pham H et al. 2015 |
| Bat | *Rhinolophus sinicus* | Rhinolophidae | Vietnam | Asia | Anh, Pham H et al. 2015 |
| Bat | *Myotis daubentonii* | Vespertilionidae | Finland | Europe | Lilley, Thomas M et al. 2015 |
| Bat | *Eptesicus nilssonii* | Vespertilionidae | Finland | Europe | Veikkolainen, Ville et al. 2014 |
| Bat | *Myotis daubentonii* | Vespertilionidae | Finland | Europe | Veikkolainen, Ville et al. 2014 |
| Bat flea | *Siphonaptera* | [Ischnopsyllidae](https://www.itis.gov/servlet/SingleRpt/SingleRpt?search_topic=TSN&search_value=152738) | Finland | Europe | Veikkolainen, Ville et al. 2014 |
| Bat fly | *Nycteribia kolenatii* | Hippoboscidae | Finland | Europe | Veikkolainen, Ville et al. 2014 |
| Bat fly | *Penicillidia monoceros* | Nycteribiidae | Finland | Europe | Veikkolainen, Ville et al. 2014 |
| Bat | *Myotis daubentonii* | Vespertilionidae | France | Europe | Stuckey, Matthew J et al. 2017 |
| Bat | *Myotis mystacinus* | Vespertilionidae | France | Europe | Stuckey, Matthew J et al. 2017 |
| Bat | *Nyctalus noctula* | Vespertilionidae | France | Europe | Stuckey, Matthew J et al. 2017 |
| Bat | *Pipistrellus nathusii* | Vespertilionidae | France | Europe | Stuckey, Matthew J et al. 2017 |
| Bat flea | *Ischnopsyllus octactenus* | Ischnopsyllidae | Hungary | Europe | Hornok, Sándor et al. 2012 |
| Bat fly | *Nycteribia* sp. | Nycteribiidae | Hungary | Europe | Hornok, Sándor et al. 2012 |
| Bat mite | *Spinturnix myoti* | Spinturnicidae | Hungary | Europe | Hornok, Sándor et al. 2012 |
| Bat mite | *Steatonyssus occidentalis* | Macronyssidae | Hungary | Europe | Hornok, Sándor et al. 2012 |
| Bat tick | *Argas vespertilionis* | Argasidae | Hungary | Europe | Hornok, Sándor et al. 2019 |
| Bat fly | *Basilia nana* | Nycteribiidae | Hungary/Romania | Europe | Sándor, Attila D et al. 2018 |
| Bat fly | *Basilia nattereri* | Nycteribiidae | Hungary/Romania | Europe | Sándor, Attila D et al. 2018 |
| Bat fly | *Nycteribia kolenatii* | Nycteribiidae | Hungary/Romania | Europe | Sándor, Attila D et al. 2018 |
| Bat fly | *Nycteribia pedicularia* | Nycteribiidae | Hungary/Romania | Europe | Sándor, Attila D et al. 2018 |
| Bat fly | *Nycteribia schmidlii* | Nycteribiidae | Hungary/Romania | Europe | Sándor, Attila D et al. 2018 |
| Bat fly | *Nycteribia vexata* | Nycteribiidae | Hungary/Romania | Europe | Sándor, Attila D et al. 2018 |
| Bat fly | *Penicillidia conspicua* | Nycteribiidae | Hungary/Romania | Europe | Sándor, Attila D et al. 2018 |
| Bat fly | *Penicillidia dufourii* | Nycteribiidae | Hungary/Romania | Europe | Sándor, Attila D et al. 2018 |
| Bat fly | *Phthiridium biarticulatum* | Nycteribiidae | Hungary/Romania | Europe | Sándor, Attila D et al. 2018 |
| Bat | *Myotis myotis* | Vespertilionidae | Poland | Europe | Szubert-Kruszyńska, Agnieszka et al. 2019 |
| Bat mite | *Spinturnix myoti* | Spinturnicidae | Poland | Europe | Szubert-Kruszyńska, Agnieszka et al. 2019 |
| Bat | *Nyctalus noctula* | Vespertilionidae | Romania | Europe | Corduneanu, Alexandra et al. 2021 |
| Bat tick | *Ixodes vespertilionis* | Ixodidae | Romania | Europe | Hornok, Sándor et al. 2019 |
| Bat fly | *Basilia nattereri* | Nycteribiidae | Slovenia | Europe | Morse, Solon F et al. 2012 |
| Bat | ND | ND | Spain | Europe | Stuckey, Matthew J et al. 2017 |
| Bat | *Myotis daubentonii* | Vespertilionidae | UK | Europe | Concannon, R et al. 2005 |
| Bat | *Myotis mystacinus* | Vespertilionidae | UK | Europe | Concannon, R et al. 2005 |
| Bat | *Nyctalus noctula* | Vespertilionidae | UK | Europe | Concannon, R et al. 2005 |
| Bat | *Pipistrellus* sp. | Vespertilionidae | UK | Europe | Concannon, R et al. 2005 |
| Bat fly | *Trichobius adamsi* | Streblidae | Dominican Republic | North America | Morse, Solon F et al. 2012 |
| Bat fly | *Trichobius frequens* | Streblidae | Puerto Rico | North America | Morse, Solon F et al. 2012 |
| Bat | *Artibeus jamaicensis* | Phyllostomidae | Saint Kitts Island | North America | Reeves, Will K et al. 2016 |
| Bat | *Ardops nichollsi* | Phyllostomidae | Saint Kitts Island | North America | Reeves, Will K et al. 2016 |
| Bat | *Brachyphylla cavernarum* | Phyllostomidae | Saint Kitts Island | North America | Reeves, Will K et al. 2016 |
| Bat mite | *Perigilischrus iheringi* | Spinturnicidae | Saint Kitts Island | North America | Reeves, Will K et al. 2016 |
| Bat | *Myotis lucifugus* | Vespertilionidae | USA | North America | Lilley, Thomas M et al. 2017 |
| Bat | *Eptesicus fuscus* | Vespertilionidae | USA | North America | McKee, Clifton D et al. 2018 |
| Bat | *Artibeus jamaicensis* | Phyllostomidae | USA | North America | Olival, Kevin J et al. 2015 |
| Bat | *Brachyphylla cavernarum* | Phyllostomidae | USA | North America | Olival, Kevin J et al. 2015 |
| Bat | *Monophyllus redmani* | Phyllostomidae | USA | North America | Olival, Kevin J et al. 2015 |
| Bat fly | *Trichobius corynorhinus* | Streblidae | USA | North America | Morse, Solon F et al. 2012 |
| Bat fly | *Trichobius major* | Streblidae | USA, | North America | Reeves, Will K et al. 2005 |
| Bat tick | *Carios kelleyi* | Argasidae | USA | North America | Loftis, Amanda D et al. 2005 |
| Bat bug | *Cimex adjunctus* | Cimicidae | USA | North America | McKee, Clifton D et al. 2018 |
| Bat flea | *Sternopsylla texanus* | Ischnopsyllidae | USA | North America | Reeves, Will K et al. 2007 |
| Bat | *Desmodus rotundus* | Phyllostomidae | Belize | Central America | Becker, [Daniel J](https://pubmed.ncbi.nlm.nih.gov/?term=Becker+DJ&cauthor_id=30260954) et al. 2018 |
| Bat | *Anoura geoffroyi* | Phyllostomidae | Costa Rica | Central America | Judson, S D et al. 2015 |
| Bat | *Artibeus jamaicensis* | Phyllostomidae | Costa Rica | Central America | Judson, S D et al. 2015 |
| Bat | *Artibeus lituratus* | Phyllostomidae | Costa Rica | Central America | Judson, S D et al. 2015 |
| Bat | *Carollia castanea* | Phyllostomidae | Costa Rica | Central America | Judson, S D et al. 2015 |
| Bat | *Carollia perspicillata* | Phyllostomidae | Costa Rica | Central America | Judson, S D et al. 2015 |
| Bat | *Carollia sowelli* | Phyllostomidae | Costa Rica | Central America | Judson, S D et al. 2015 |
| Bat | *Micronycteris microtus* | Phyllostomidae | Costa Rica | Central America | Judson, S D et al. 2015 |
| Bat | *Myotis keaysi* | Vespertilionidae | Costa Rica | Central America | Judson, S D et al. 2015 |
| Bat | *Phyllostomus discolor* | Phyllostomidae | Costa Rica | Central America | Judson, S D et al. 2015 |
| Bat | *Platyrrhinus vittatus* | Phyllostomidae | Costa Rica | Central America | Judson, S D et al. 2015 |
| Bat | *Sturnira lilium* | Phyllostomidae | Costa Rica | Central America | Judson, S D et al. 2015 |
| Bat | *Sturnira mordax* | Phyllostomidae | Costa Rica | Central America | Judson, S D et al. 2015 |
| Bat | *Vampyressa thyone* | Phyllostomidae | Costa Rica | Central America | Judson, S D et al. 2015 |
| Bat fly | *Anastrebla modestini* | Streblidae | Costa Rica | Central America | Judson, S D et al. 2015 |
| Bat fly | *Anatrichobius scorzai* | Streblidae | Costa Rica | Central America | Judson, S D et al. 2015 |
| Bat fly | *Aspidoptera delatorrei* | Streblidae | Costa Rica | Central America | Judson, S D et al. 2015 |
| Bat fly | *Aspidoptera phyllostomatis* | Streblidae | Costa Rica | Central America | Judson, S D et al. 2015 |
| Bat fly | *Basilia* sp. | Nycteribiidae | Costa Rica | Central America | Judson, S D et al. 2015 |
| Bat fly | *Megistopoda proxima* | Streblidae | Costa Rica | Central America | Judson, S D et al. 2015 |
| Bat fly | *Neotrichobius* sp. | Streblidae | Costa Rica | Central America | Judson, S D et al. 2015 |
| Bat fly | *Paratrichobius dunni* | Streblidae | Costa Rica | Central America | Judson, S D et al. 2015 |
| Bat fly | *Paratrichobius longicrus* | Streblidae | Costa Rica | Central America | Judson, S D et al. 2015 |
| Bat fly | *Strebla guajiro* | Streblidae | Costa Rica | Central America | Judson, S D et al. 2015 |
| Bat fly | *Trichobius costalimai* | Streblidae | Costa Rica | Central America | Judson, S D et al. 2015 |
| Bat fly | *Trichobius dugesii* | Streblidae | Costa Rica | Central America | Judson, S D et al. 2015 |
| Bat fly | *Trichobius joblingi* | Streblidae | Costa Rica | Central America | Judson, S D et al. 2015 |
| Bat fly | *Trichobius keenani* | Streblidae | Costa Rica | Central America | Judson, S D et al. 2015 |
| Bat fly | *Trichobius* sp. | Streblidae | Costa Rica | Central America | Judson, S D et al. 2015 |
| Bat fly | *Joblingia schmidtii* | Streblidae | Costa Rica | Central America | Morse, Solon F et al. 2012 |
| Bat | *Artibeus toltecus* | Phyllostomidae | Guatemala | Central America | Bai, Ying et al. 2011 |
| Bat | *Carollia perspicillata* | Phyllostomidae | Guatemala | Central America | Bai, Ying et al. 2011 |
| Bat | *Desmodus rotundus* | Phyllostomidae | Guatemala | Central America | Bai, Ying et al. 2011 |
| Bat | *Glossophaga soricina* | Phyllostomidae | Guatemala | Central America | Bai, Ying et al. 2011 |
| Bat | *Micronycteris microtis* | Phyllostomidae | Guatemala | Central America | Bai, Ying et al. 2011 |
| Bat | *Phyllostomus discolor* | Phyllostomidae | Guatemala | Central America | Bai, Ying et al. 2011 |
| Bat | *Pteronotus davyi* | Mormoopidae | Guatemala | Central America | Bai, Ying et al. 2011 |
| Bat | *Sturnira lilium* | Phyllostomidae | Guatemala | Central America | Bai, Ying et al. 2011 |
| Bat | *Desmodus rotundus* | Phyllostomidae | Guatemala | Central America | Wray, Amy K et al. 2017 |
| Bat fly | *Strebla mirabilis* | Streblidae | Mexico | Central America | Morse, Solon F et al. 2012 |
| Bat fly | *Trichobius johnsonae* | Streblidae | Mexico | Central America | Morse, Solon F et al. 2012 |
| Bat fly | *Paratrichobius longicrus* | Streblidae | Mexico | Central America | Moskaluk Alexandra E et al. 2018 |
| Bat fly | *Strebla wiedemanni* | Streblidae | Mexico | Central America | Moskaluk Alexandra E et al. 2018 |
| Bat fly | *Trichobius parasiticus* | Streblidae | Mexico | Central America | Moskaluk Alexandra E et al. 2018 |
| Bat | *Artibeus jamaicensis* | Phyllostomidae | Mexico | Central America | Stuckey, Matthew J et al. 2017 |
| Bat | *Balantiopteryx plicata* | Emballonuridae | Mexico | Central America | Stuckey, Matthew J et al. 2017 |
| Bat | *Desmodus rotundus* | Phyllostomidae | Mexico | Central America | Stuckey, Matthew J et al. 2017 |
| Bat | *Pteronotus parnellii* | Mormoopidae | Mexico | Central America | Stuckey, Matthew J et al. 2017 |
| Bat | *Sturnira* spp. | Phyllostomidae | Mexico | Central America | Stuckey, Matthew J et al. 2017 |
| Bat fly | *Paradyschiria lineata* | Streblidae | Panama | Central America | Morse, Solon F et al. 2012 |
| Bat fly | *Strebla diaemi* | Streblidae | Panama | Central America | Morse, Solon F et al. 2012 |
| Bat | *Tadarida brasiliensis* | Molossidae | Argentina | South America | Cicuttin, Gabriel L et al. 2017 |
| Bat | *Desmodus rotundus* | Phyllostomidae | Brazil | South America | André, Marcos R et al. 2019 |
| Bat | *Diphylla ecaudata* | Phyllostomidae | Brazil | South America | André, Marcos R et al. 2019 |
| Bat | *Trichobius* spp. | Hippoboscidae | Brazil | South America | Braga, M et al. 2020 |
| Bat | *Artibeus fimbriatus* | Phyllostomidae | Brazil | South America | Ferreira, Michelle S et al. 2018 |
| Bat | *Artibeus lituratus* | Phyllostomidae | Brazil | South America | Ferreira, Michelle S et al. 2018 |
| Bat | *Artibeus obscurus* | Phyllostomidae | Brazil | South America | Ferreira, Michelle S et al. 2018 |
| Bat | *Carollia perspicillata* | Phyllostomidae | Brazil | South America | Ferreira, Michelle S et al. 2018 |
| Bat | *Desmodus rotundus* | Phyllostomidae | Brazil | South America | Ferreira, Michelle S et al. 2018 |
| Bat | *Phyllostomus discolor* | Phyllostomidae | Brazil | South America | Ferreira, Michelle S et al. 2018 |
| Bat | *Rhinophylla pumilio* | Phyllostomidae | Brazil | South America | Ferreira, Michelle S et al. 2018 |
| Bat | *Sturnira lilium* | Phyllostomidae | Brazil | South America | Ferreira, Michelle S et al. 2018 |
| Bat | *Artibeus lituratus* | Phyllostomidae | Brazil | South America | Gonçalves-Oliveira, Jonathan et al. 2020 |
| Bat | *Carollia perspicillata* | Phyllostomidae | Brazil | South America | Gonçalves-Oliveira, Jonathan et al. 2020 |
| Bat | *Carollia perspicillata* | Phyllostomidae | Brazil | South America | Ikeda, Priscila et al. 2017 |
| Bat | *Glossophaga soricina* | Phyllostomidae | Brazil | South America | Ikeda, Priscila et al. 2017 |
| Bat | *Myotis molossus* | Vespertilionidae | Brazil | South America | Ikeda, Priscila et al. 2017 |
| Bat | *Myotis nigricans* | Vespertilionidae | Brazil | South America | Ikeda, Priscila et al. 2017 |
| Bat | *Myotis rufus* | Vespertilionidae | Brazil | South America | Ikeda, Priscila et al. 2017 |
| Bat | *phyllostomus discolor* | Phyllostomidae | Brazil | South America | Ikeda, Priscila et al. 2017 |
| Bat | *Sturnira lilium* | Phyllostomidae | Brazil | South America | Ikeda, Priscila et al. 2017 |
| Bat | *Artibeus planirostris* | Phyllostomidae | Brazil | South America | Ikeda, Priscila et al. 2020 |
| Bat | *Carollia perspicillata* | Phyllostomidae | Brazil | South America | Ikeda, Priscila et al. 2020 |
| Bat | *Platyrrhinus lineatus* | Phyllostomidae | Brazil | South America | Ikeda, Priscila et al. 2020 |
| Bat fly | *Megistopoda aranea* | Hippoboscidae | Brazil | South America | Ikeda, Priscila et al. 2020 |
| Bat fly | *Trichobius costalimai* | Streblidae | Brazil | South America | Ikeda, Priscila et al. 2020 |
| Bat fly | *Trichobius dugesii* complex | Streblidae | Brazil | South America | Ikeda, Priscila et al. 2020 |
| Bat fly | *Aspidoptera falcata* | Streblidae | Brazil | South America | do Amaral, Renan B et al. 2018 |
| Bat fly | *Aspidoptera phyllostomatis* | Streblidae | Brazil | South America | do Amaral, Renan B et al. 2018 |
| Bat fly | *Megistopoda aranea* | Streblidae | Brazil | South America | do Amaral, Renan B et al. 2018 |
| Bat fly | *Paratrichobius longicrus* | Streblidae | Brazil | South America | do Amaral, Renan B et al. 2018 |
| Bat fly | *Strebla guajiro* | Streblidae | Brazil | South America | do Amaral, Renan B et al. 2018 |
| Bat fly | *Trichobius joblingi* | Streblidae | Brazil | South America | do Amaral, Renan B et al. 2018 |
| Bat fly | *Megistopoda aranea* | Hippoboscidae | Brazil | South America | do Amaral, Renan B et al. 2018 |
| Bat fly | *Streblaguajiro* | Streblidae | Brazil | South America | do Amaral, Renan B et al. 2018 |
| Bat fly | *Trichobius joblingi* | Streblidae | Brazil | South America | do Amaral, Renan B et al. 2018 |
| Bat mite | *Steatonyssus* spp. | Macronyssidae | Brazil | South America | Ikeda, Priscila et al. 2020 |
| Bat | *Histiotis montanus* | Vespertilionidae | Chile | South America | Müller, Ananda et al. 2020 |
| Bat | *Myotis chiloensis* | Vespertilionidae | Chile | South America | Müller, Ananda et al. 2020 |
| Bat | *Tadarida brasiliensis* | Molossidae | Chile | South America | Müller, Ananda et al. 2020 |
| Bat | *Noctilio albiventris* | Noctilionidae | French Guiana | South America | Davoust, Bernard et al. 2016 |
| Bat | *Pteronotus parnellii* | Mormoopidae | French Guiana | South America | Davoust, Bernard et al. 2016 |
| Bat fly | *Paratrichobius longicrus* complex | Streblidae | French Guiana | South America | Morse, Solon F et al. 2012 |
| Bat tick | *Ornithodoros hasei* | Argasidae | French Guiana | South America | Davoust, Bernard et al. 2016 |
| Bat | *Artibeus obscurus* | Phyllostomidae | Peru | South America | Bai, Ying et al. 2012 |
| Bat | *Artibeus planirostris* | Phyllostomidae | Peru | South America | Bai, Ying et al. 2012 |
| Bat | *Carollia brevicauda* | Phyllostomidae | Peru | South America | Bai, Ying et al. 2012 |
| Bat | *Carollia perspicillata* | Phyllostomidae | Peru | South America | Bai, Ying et al. 2012 |
| Bat | *Desmodus rotundus* | Phyllostomidae | Peru | South America | Bai, Ying et al. 2012 |
| Bat | *Glossophaga soricina* | Phyllostomidae | Peru | South America | Bai, Ying et al. 2012 |
| Bat | *Myotis* sp. | Vespertilionidae | Peru | South America | Bai, Ying et al. 2012 |
| Bat | *Phyllostomus discolor* | Phyllostomidae | Peru | South America | Bai, Ying et al. 2012 |
| Bat | *Phyllostomus hastatus* | Phyllostomidae | Peru | South America | Bai, Ying et al. 2012 |
| Bat | *Sturnira lilium* | Phyllostomidae | Peru | South America | Bai, Ying et al. 2012 |
| Bat | *Vampyricus bidens* | Phyllostomidae | Peru | South America | Bai, Ying et al. 2012 |
| Bat | *Desmodus rotundus* | Phyllostomidae | Peru | South America | Becker, [Daniel J](https://pubmed.ncbi.nlm.nih.gov/?term=Becker+DJ&cauthor_id=30260954) et al. 2018 |
| Bat fly | *Pseudostrebla riberoi* | Streblidae | Peru | South America | Morse, Solon F et al. 2012 |

Search strategy: (bartonella OR bartonellae) AND (bat OR bats) in Pubmed.
