# Supplementary material for: Bats and their ectoparasites (Nycteribiidae and Spinturnicidae) carry diverse novel Bartonella genotypes, China
Source: Transbound Emerg Dis. 2021 Nov 2;69(4):e845–58. doi: 10.1111/tbed.14357 (PMC9543326; doi:10.1111/tbed.14357)
Supplement: Supplementary file 3 — Table S2 Primers used in this study [file TBED-69-e845-s003.docx]

Table S2. Primers used in this study.

| Target species | Gene | Primers | Sequences (5′-3′ ) | PCR products (bp) | Reference |
| --- | --- | --- | --- | --- | --- |
| *Bartonella* | *gltA* | gltA-F | GCTATGTCTGCATTCTATCA | 751 | (1) |
|  |  | gltA-R | GATCYTCAATCATTTCTTTCCA |  |  |
|  |  | gltA-F1 | GCTATGTCTGCVTTCTATCAYGA | 731 | (2) |
|  |  | gltA-OR1 | AGAACAGTAAACATTTCNGTHGG |  |  |
|  | *rpoB* | rpoB-F | CGCATTGGCTTACTTCGTATG | 852 | (1) |
|  |  | rpoB-R | GTAGACTGATTAGAACGCTG |  |  |
|  | *ftsZ* | ftsZ-F | ATTAATCTGCAYCGGCCAGA | 885 | (1) |
|  |  | ftsZ-R | ACVGADACACGAATAACACC |  |  |
|  |  | ftsZ-F1 | ATTAATCTGCAYCGGCCAGATAT | 791 | (2) |
|  |  | ftsZ-R1 | TCATCAATRGCVCCAAARAT |  |  |
| Bat | *cytB* | cytB-F | CCATGAGGCCAAATATCCTTCTGAGG | 604 | (3) |
|  |  | cytB-R | TTGGCCAATGATAATGTAKGGRTGTTC |  |  |
| Bat fly | *COI* | LCO1490 | GGTCAACAAATCATAAAGATATTGG | 658 | (4) |
|  |  | HCO2198 | TAAACTTCAGGGTGACCAAAAAATCA |  |  |
|  | 16S rRNA | 16S-F | CCAAAAAATTATTTTAATCCAACATCGAGG | 460 | (5) |
|  |  | 16S-R | CACCTGTTTATCAAAAACAT |  |  |
| Bat mite | *COI* | C1-J-2183 | CAACATTTATTTTGATTTTTTGG | 658 | (6) |
|  |  | C1-J-2797 mod | GGATAATCTGAATAACGTCGAGG |  |  |
|  | 16s rRNA | 16s+1 | CCGGTCTGAACTCAGATCAAGT | 543 | (6) |
|  |  | 16s-1 | GCTCAATGATTTTTTAAATTGCTGT |  |  |

**References**

1. Bai Y, Hayman DT, McKee CD, Kosoy MY. Classification of *Bartonella* strains associated with straw-colored fruit bats (*Eidolon helvum*) across Africa using a multi-locus sequence typing platform. PLoS Negl Trop Dis. 2015 Jan;9(1):e0003478.

2. Gonçalves-Oliveira J, Rozental T, Guterres A, Teixeira BR, Andrade-Silva BE, Costa-Neto SFD, et al. Investigation of *Bartonella* spp. in brazilian mammals with emphasis on rodents and bats from the Atlantic Forest. Int J Parasitol Parasites Wildl. 2020 Dec;13:80-9.

3. Li ZM, Xiao X, Zhou CM, Liu JX, Gu XL, Fang LZ, et al. Human-pathogenic relapsing fever *Borrelia* found in bats from Central China phylogenetically clustered together with relapsing fever borreliae reported in the New World. PLoS Negl Trop Dis. 2021 Mar;15(3):e0009113.

4. Folmer O, Black M, Hoeh W, Lutz R, Vrijenhoek R. DNA primers for amplification of mitochondrial cytochrome c oxidase subunit I from diverse metazoan invertebrates. Mol Mar Biol Biotechnol. 1994 Oct;3(5):294-9.

5. Castro LR, Austin AD, Dowton M. Contrasting Rates of Mitochondrial Molecular Evolution in Parasitic Diptera and Hymenoptera. Molecular Biology and Evolution. 2002;19(7):1100-13.

6. Bruyndonckx N, Dubey S, Ruedi M, Christe P. Molecular cophylogenetic relationships between European bats and their ectoparasitic mites (Acari, Spinturnicidae). Mol Phylogenet Evol. 2009 May;51(2):227-37.
