# Supplementary material for: Bats and their ectoparasites (Nycteribiidae and Spinturnicidae) carry diverse novel Bartonella genotypes, China
Source: Transbound Emerg Dis. 2021 Nov 2;69(4):e845–58. doi: 10.1111/tbed.14357 (PMC9543326; doi:10.1111/tbed.14357)
Supplement: Supplementary file 4 — Table S3 Detailed information on Bartonella‐positive samples of this study [file TBED-69-e845-s001.docx]

Table S3. Detailed information on *Bartonella*-positive samples of this study.

| Sampling date | Sampling area | Sample type | Sample species | Sample ID | *gltA* | *rpoB* | *ftsZ* |
| --- | --- | --- | --- | --- | --- | --- | --- |
| 2018 | Songzi | Bat | *Myotis adversus* | 38 | - | - | MZ208728 |
|  |  |  | *Myotis adversus* | 62 | MZ208699 (Ⅶ) | - | MZ208729 |
|  |  | Bat fly | *Penicillidia monoceros* | 30-1 | + | + | MZ208703 |
|  |  |  | *Penicillidia monoceros* | 30-3 | MZ208681 (Ⅶ) | MZ208739 (Ⅶ) | MZ208703 |
|  |  |  | *Penicillidia monoceros* | 35-2 | + | + | MZ208703 |
|  |  |  | *Penicillidia monoceros* | 35-3 | MZ208681 (Ⅶ) | MZ208739 (Ⅶ) | MZ208703 |
|  |  |  | *Penicillidia monoceros* | 38 | + | + | MZ208703 |
|  |  |  | *Penicillidia monoceros* | 40 | + | + | MZ208703 |
|  |  |  | *Penicillidia monoceros* | 43 | MZ208681 (Ⅶ) | MZ208739 (Ⅶ) | MZ208703 |
|  |  |  | *Penicillidia monoceros* | 44 | + | + | MZ208703 |
|  |  |  | *Penicillidia monoceros* | 59-2 | MZ208681 (Ⅶ) | MZ208739 (Ⅶ) | MZ208705 |
|  |  |  | *Penicillidia monoceros* | 62 | + | + | MZ208703 |
|  |  | Bat fly^*^ | *Nycteribia* sp. | 52 | MZ208682 (Ⅵ) | MZ208740 (Ⅹ) | MZ208704 |
| 2019 | Jingzhou | Bat | *Myotis davidii* | 002 | - | - | MZ208730 |
|  |  |  | *Myotis davidii* | 115 | MZ208700 (Ⅳ) | - | - |
|  |  |  | *Myotis davidii* | 194 | - | MZ208763 (Ⅲ) | MZ208731 |
|  |  | Bat fly^*^ | *Penicillidia monoceros*  *Nycteribia*sp.  *Phthiridium*sp. | 1 | MZ208683 (Ⅶ) | MZ208744 (Ⅶ) | MZ208708 |
|  |  |  |  | 2 | MZ208683 (Ⅶ) | MZ208744 (Ⅶ) | MZ208708 |
|  |  |  |  | 3 | MZ208683 (Ⅶ) | MZ208744 (Ⅶ) | MZ208708 |
|  |  |  |  | 4 | MZ208686(Ⅲ) MZ208683 (Ⅶ) | MZ208741 (Ⅺ)  MZ208744 (Ⅶ) | MZ208708  MZ208706  MZ208707 |
|  |  |  |  | 5 | MZ208687(Ⅰ) MZ208683 (Ⅶ) | MZ208744 (Ⅶ) | MZ208708  MZ208709 |
|  |  |  |  | 6 | MZ208683 (Ⅶ) | MZ208742 (Ⅴ) | MZ208710 |
|  |  |  |  | 7 | MZ208685(Ⅵ) MZ208683 (Ⅶ) | MZ208743 (Ⅳ)  MZ208744 (Ⅶ) | MZ208708 |
|  |  |  |  | 8 | MZ208683 (Ⅶ) | MZ208745(Ⅹ) MZ208744 (Ⅶ) | MZ208710 |
|  |  |  |  | 9 | MZ208683 (Ⅶ) | MZ208744 (Ⅶ) | MZ208708 |
|  |  |  |  | 10 | MZ208686 (Ⅲ) | MZ208746(Ⅵ) MZ208742 (Ⅴ) | MZ208710MZ208711  MZ208712 |
|  |  |  |  | 11 | MZ208683 (Ⅶ) | MZ208747 (Ⅷ) | MZ208713  MZ208714 |
|  |  |  |  | 12 | MZ208683 (Ⅶ) | MZ208744(Ⅶ) MZ208741 (Ⅺ) | MZ208708 |
|  |  |  |  | 13 | MZ208684 (Ⅵ) | MZ208744 (Ⅶ) | MZ208708  MZ208715  MZ208716 |
|  |  |  |  | 14 | MZ208683 (Ⅶ) | MZ208748 (Ⅶ) | MZ208708 |
|  |  |  |  | 15 | MZ208688 (Ⅴ) | MZ208749(Ⅸ) MZ208751 (Ⅲ) | MZ208717 |
|  |  |  |  | 16 | MZ208688 (Ⅴ)  MZ208683 (Ⅶ) | MZ208750 (Ⅱ)  MZ208751(Ⅲ) MZ208741 (Ⅺ) | MZ208715  MZ208718  MZ208719 |
| 2020 | Xianning | Bat | *Rhinolophus pusillus* | 707 | - | - | MZ208732 |
|  |  |  | *Myotis davidii* | 723 | MZ208702 (Ⅰ) | MZ208765 (Ⅺ) | MZ208734 |
|  |  |  | *Myotis davidii* | 732 | - | MZ208765 (Ⅺ) | MZ208734 |
|  |  |  | *Myotis davidii* | 768 | - | MZ208765 (Ⅺ) | - |
|  |  |  | *Myotis davidii* | 789 | - | MZ208765 (Ⅺ) | - |
|  |  |  | *Myotis davidii* | 794 | MZ208702 (Ⅰ) | MZ208765 (Ⅺ) | MZ208734 |
|  |  |  | *Rhinolophus pusillus* | 796 | MZ208701 (Ⅳ) | MZ208766 (Ⅴ) | - |
|  |  |  | *Myotis davidii* | 798 | - | MZ208764 (ⅩⅢ) | - |
|  |  |  | *Myotis davidii* | 800 | - | - | MZ208734 |
|  |  |  | *Rhinolophus pusillus* | 817 | - | MZ208765 (Ⅺ) | - |
|  |  |  | *Myotis davidii* | 820 | MZ208702 (Ⅰ) | MZ208765 (Ⅺ) | MZ208734 |
|  |  |  | *Myotis davidii* | 822 | - | - | MZ208734 |
|  |  |  | *Myotis davidii* | 825 | - | - | MZ208733 |
|  |  |  | *Myotis davidii* | 831 | - | - | MZ208735 |
|  |  |  | *Myotis adversus* | 833 | MZ208702 (Ⅰ) | MZ208765 (Ⅺ) | MZ208734 |
|  |  |  | *Myotis davidii* | 841 | MZ208702 (Ⅰ) | MZ208765 (Ⅺ) | MZ208733 |
|  |  |  | *Myotis davidii* | 844 | - | MZ208765 (Ⅺ) | MZ208734 |
|  |  |  | *Myotis davidii* | 849 | - | MZ208765 (Ⅺ) | MZ208734 |
|  |  |  | *Myotis davidii* | 859 | - | MZ208765 (Ⅺ) | - |
|  |  |  | *Myotis davidii* | 877 | MZ208702 (Ⅰ) | - | MZ208734 |
|  |  |  | *Myotis davidii* | 902 | MZ208702 (Ⅰ) | MZ208765 (Ⅺ) | MZ208735 |
|  |  | Bat fly | *Penicillidia monoceros* | 1-1 | MZ208690 (Ⅶ) | + | + |
|  |  |  | *Penicillidia monoceros* | 1-3 | MZ208690 (Ⅶ) | + | + |
|  |  |  | *Penicillidia monoceros* | 1-4 | MZ208690 (Ⅶ) | + | + |
|  |  |  | *Penicillidia monoceros* | 1-6 | MZ208690 (Ⅶ) | + | + |
|  |  |  | *Penicillidia monoceros* | 1-8 | MZ208690 (Ⅶ) | MZ208752 (Ⅶ) | MZ208721 |
|  |  |  | *Penicillidia monoceros* | 1-9 | MZ208690 (Ⅶ) | + | + |
|  |  |  | *Penicillidia monoceros* | 1-10 | MZ208690 (Ⅶ) | + | + |
|  |  |  | *Penicillidia monoceros* | 1-11 | MZ208690 (Ⅶ) | + | + |
|  |  |  | *Penicillidia monoceros* | 1-12 | MZ208689 (Ⅰ) | MZ208753 (Ⅺ) | MZ208720 |
|  |  |  | *Penicillidia monoceros* | 1-13 | MZ208690 (Ⅶ) | + | + |
|  |  |  | *Penicillidia monoceros* | 1-14 | MZ208690 (Ⅶ) | + | + |
|  |  |  | *Penicillidia monoceros* | 1-16 | MZ208690 (Ⅶ) | + | + |
|  |  |  | *Penicillidia monoceros* | 1-17 | MZ208690 (Ⅶ) | + | + |
|  |  |  | *Penicillidia monoceros* | 1-18 | MZ208690 (Ⅶ) | + | + |
|  |  |  | *Penicillidia monoceros* | 1-19 | MZ208690 (Ⅶ) | + | + |
|  |  |  | *Penicillidia monoceros* | 1-20 | MZ208690 (Ⅶ) | + | + |
|  |  |  | *Penicillidia monoceros* | 1-21 | MZ208690 (Ⅶ) | + | + |
|  |  |  | *Penicillidia monoceros* | 1-22 | MZ208690 (Ⅶ) | + | + |
|  |  |  | *Penicillidia monoceros* | 1-23 | MZ208690 (Ⅶ) | + | + |
|  |  |  | *Penicillidia monoceros* | 1-24 | MZ208690 (Ⅶ) | + | + |
|  |  |  | *Penicillidia monoceros* | 2-1 | MZ208690 (Ⅶ) | + | + |
|  |  |  | *Penicillidia monoceros* | 2-2 | MZ208690 (Ⅶ) | MZ208752 (Ⅶ) | MZ208721 |
|  |  |  | *Penicillidia monoceros* | 2-3 | MZ208690 (Ⅶ) | + | + |
|  |  |  | *Penicillidia monoceros* | 2-11 | MZ208690 (Ⅶ) | + | + |
|  |  |  | *Penicillidia monoceros* | 2-12 | MZ208690 (Ⅶ) | + | + |
|  |  |  | *Penicillidia monoceros* | 2-13 | MZ208690 (Ⅶ) | + | + |
|  |  |  | *Penicillidia monoceros* | 2-14 | MZ208690 (Ⅶ) | + | + |
|  |  |  | *Penicillidia monoceros* | 2-15 | MZ208690 (Ⅶ) | + | + |
|  |  |  | *Penicillidia monoceros* | 2-16 | MZ208690 (Ⅶ) | + | + |
|  |  |  | *Penicillidia monoceros* | 2-17 | MZ208690 (Ⅶ) | + | + |
|  |  |  | *Penicillidia monoceros* | 2-18 | MZ208690 (Ⅶ) | + | + |
|  |  |  | *Penicillidia monoceros* | 2-21 | MZ208690 (Ⅶ) | + | + |
|  |  |  | *Penicillidia monoceros* | 2-25 | MZ208690 (Ⅶ) | + | + |
|  |  |  | *Penicillidia monoceros* | 2-26 | MZ208690 (Ⅶ) | + | + |
|  |  |  | *Penicillidia monoceros* | 2-27 | MZ208690 (Ⅶ) | + | + |
|  |  |  | *Penicillidia monoceros* | 2-28 | MZ208690 (Ⅶ) | + | + |
|  |  |  | *Penicillidia monoceros* | 2-29 | MZ208690 (Ⅶ) | + | + |
|  |  |  | *Penicillidia monoceros* | 2-30 | MZ208690 (Ⅶ) | + | + |
|  |  |  | *Penicillidia monoceros* | 2-33 | MZ208690 (Ⅶ) | + | + |
|  |  |  | *Penicillidia monoceros* | 2-34 | MZ208690 (Ⅶ) | + | + |
|  |  |  | *Penicillidia monoceros* | 2-35 | MZ208690 (Ⅶ) | + | + |
|  |  |  | *Penicillidia monoceros* | 3-2 | MZ208690 (Ⅶ) | + | + |
|  |  |  | *Penicillidia monoceros* | 3-10 | MZ208690 (Ⅶ) | MZ208752 (Ⅶ) | MZ208721 |
|  |  |  | *Penicillidia monoceros* | 3-11 | MZ208689 (Ⅰ) | MZ208753 (Ⅺ) | MZ208720 |
|  |  |  | *Penicillidia monoceros* | 4-6 | MZ208690 (Ⅶ) | + | + |
|  |  |  | *Penicillidia monoceros* | 4-7 | MZ208690 (Ⅶ) | + | + |
|  |  |  | *Penicillidia monoceros* | 4-12 | MZ208690 (Ⅶ) | + | + |
|  |  |  | *Penicillidia monoceros* | 4-14 | MZ208689 (Ⅰ) | + | + |
|  |  |  | *Penicillidia monoceros* | 4-22 | MZ208690 (Ⅶ) | + | + |
|  |  |  | *Penicillidia monoceros* | 4-23 | MZ208690 (Ⅶ) | + | + |
|  |  |  | *Penicillidia monoceros* | 4-26 | MZ208690 (Ⅶ) | + | + |
|  |  |  | *Penicillidia monoceros* | 4-28 | MZ208690 (Ⅶ) | + | + |
|  |  |  | *Penicillidia monoceros* | 4-31 | MZ208690 (Ⅶ) | + | + |
|  |  |  | *Penicillidia monoceros* | 4-35 | MZ208690 (Ⅶ) | + | + |
|  |  |  | *Penicillidia monoceros* | 4-38 | MZ208690 (Ⅶ) | + | + |
|  |  |  | *Penicillidia monoceros* | 4-41 | MZ208690 (Ⅶ) | + | + |
|  |  |  | *Penicillidia monoceros* | 4-42 | MZ208690 (Ⅶ) | + | + |
|  |  |  | *Penicillidia monoceros* | 4-43 | MZ208690 (Ⅶ) | + | + |
|  |  |  | *Penicillidia monoceros* | 4-44 | MZ208690 (Ⅶ) | MZ208752 (Ⅶ) | MZ208721 |
|  |  |  | *Penicillidia monoceros* | 4-46 | MZ208690 (Ⅶ) | + | + |
|  |  | Bat fly^*^ | *Nycteribia* sp.  *Phthiridium* sp. | 1 | MZ208691 (Ⅶ) | MZ208757(Ⅵ) MZ208754 (Ⅹ) | MZ208722 |
|  |  |  |  | 2 | MZ208692(Ⅵ) MZ208691 (Ⅶ) | MZ208755 (Ⅹ) | MZ208724 |
|  |  |  |  | 3 | MZ208694 (Ⅵ) | MZ208757 (Ⅵ) | MZ208722 |
|  |  |  |  | 4 | MZ208693 (Ⅱ)  MZ208694 (Ⅵ) | MZ208757 (Ⅵ) | MZ208722 |
|  |  |  |  | 5 | MZ208695 (Ⅲ) | MZ208757 (Ⅵ) | MZ208722  MZ208723 |
|  |  |  |  | 6 | MZ208695 (Ⅲ) | MZ208754 (Ⅹ) | MZ208723 |
|  |  | Bat mite^*^ | *Spinturnix* sp.  *Eyndhovenia* sp. | 1 | MZ208696 (Ⅴ) | MZ208758 (Ⅲ) | MZ208725 |
|  |  |  |  | 2 | MZ208698 (Ⅳ) | MZ208759 (ⅩⅢ) | MZ208727 |
|  |  |  |  | 3 | MZ208698 (Ⅳ) | MZ208762(Ⅰ) MZ208760 (Ⅺ) | MZ208725 |
|  |  |  |  | 4 | MZ208698 (Ⅳ) | MZ208761 (Ⅻ) | MZ208727 |
|  |  |  |  | 5 | MZ208696 (Ⅴ) | MZ208762(Ⅰ) MZ208760 (Ⅺ) | MZ208725 |
|  |  |  |  | 6 | MZ208696 (Ⅴ) | MZ208760 (Ⅺ) | MZ208726 |
|  |  |  |  | 7 | MZ208697(Ⅰ) MZ208698 (Ⅳ) | MZ208760 (Ⅺ) | MZ208725 |
|  |  |  |  | 8 | MZ208698 (Ⅳ) | MZ208761 (Ⅻ) | MZ208727 |
|  |  |  |  | 9 | MZ208696 (Ⅴ) | MZ208761 (Ⅻ) | MZ208727 |
|  |  |  |  | 10 | MZ208698(Ⅳ) MZ208696 (Ⅴ) | MZ208762(Ⅰ) MZ208760 (Ⅺ) | MZ208725 |
|  |  |  |  | 11 | MZ208696 (Ⅴ)  MZ208698 (Ⅳ) | MZ208760 (Ⅺ)  MZ208761 (Ⅻ) | MZ208727 |

^*^ indicated pooled samples.

- and + represented negative PCR results and positive PCR results without sequencing, respectively.

The Roman numerals in the parenthesis corresponded to the *Bartonella* species defined based on the *gltA* and *rpoB* genes in Figure 5 and 6.
