# Supplementary material for: Bats and their ectoparasites (Nycteribiidae and Spinturnicidae) carry diverse novel Bartonella genotypes, China
Source: Transbound Emerg Dis. 2021 Nov 2;69(4):e845–58. doi: 10.1111/tbed.14357 (PMC9543326; doi:10.1111/tbed.14357)
Supplement: Supplementary file 5 — Table S4 Estimates of evolutionary divergence between Bartonella mayotimonensis and closely related bat‐borne Bartonella species based on the gltA gene (327 bp) [file TBED-69-e845-s005.docx]

Table S4. Estimates of evolutionary divergence between *Bartonella mayotimonensis* and closely related bat-borne *Bartonella* species based on the *gltA* gene (327 bp).

|  | 1 | 2 | 3 | 4 | 5 | 6 | 7 | 8 | 9 | 10 | 11 | 12 | 13 | 14 | 15 |
| --- | --- | --- | --- | --- | --- | --- | --- | --- | --- | --- | --- | --- | --- | --- | --- |
| 1 FJ376732*Candidatus* Bartonella mayotimonensis |  |  |  |  |  |  |  |  |  |  |  |  |  |  |  |
| 2 MZ208693 *Bartonella* sp. (Bat fly) | 0.081 |  |  |  |  |  |  |  |  |  |  |  |  |  |  |
| 3 MZ208687 *Bartonella* sp. (Bat fly) | 0.085 | 0.051 |  |  |  |  |  |  |  |  |  |  |  |  |  |
| 4 MZ208689 *Bartonella* sp. (Bat fly) | 0.092 | 0.051 | 0.006 |  |  |  |  |  |  |  |  |  |  |  |  |
| 5 MZ208697 *Bartonella* sp. (Bat mite) | 0.092 | 0.051 | 0.006 | 0.006 |  |  |  |  |  |  |  |  |  |  |  |
| 6 MZ208702 *Bartonella* sp. (Bat) | 0.099 | 0.058 | 0.012 | 0.012 | 0.012 |  |  |  |  |  |  |  |  |  |  |
| 7 KX655815 *Bartonella* sp. (Bat Shandong, China) | 0.081 | 0.048 | 0.022 | 0.022 | 0.022 | 0.028 |  |  |  |  |  |  |  |  |  |
| 8 KX807177 *Bartonella* sp. (Bat the USA) | 0.003 | 0.085 | 0.082 | 0.089 | 0.089 | 0.096 | 0.078 |  |  |  |  |  |  |  |  |
| 9 AJ871615 *Bartonella* sp. (Bat the UK) | 0.074 | 0.054 | 0.051 | 0.051 | 0.051 | 0.051 | 0.047 | 0.078 |  |  |  |  |  |  |  |
| 10 KF003137 *Bartonella* sp. (Bat Finland) | 0.074 | 0.038 | 0.038 | 0.038 | 0.038 | 0.044 | 0.028 | 0.078 | 0.034 |  |  |  |  |  |  |
| 11 KY041991 *Bartonella* sp.(Bat France) | 0.077 | 0.056 | 0.056 | 0.056 | 0.056 | 0.056 | 0.053 | 0.081 | 0.003 | 0.039 |  |  |  |  |  |
| 12 KY041985 *Bartonella* sp. (Bat France) | 0.085 | 0.057 | 0.033 | 0.033 | 0.033 | 0.040 | 0.023 | 0.082 | 0.053 | 0.033 | 0.053 |  |  |  |  |
| 13 AJ871613 *Bartonella* sp. (Bat the UK) | 0.085 | 0.058 | 0.031 | 0.031 | 0.031 | 0.038 | 0.022 | 0.081 | 0.051 | 0.031 | 0.056 | 0.000 |  |  |  |
| 14 KF003122 *Bartonella* sp. (Bat Finland) | 0.085 | 0.058 | 0.031 | 0.031 | 0.031 | 0.038 | 0.022 | 0.081 | 0.051 | 0.031 | 0.056 | 0.000 | 0.000 |  |  |
| 15 KY041981 *Bartonella* sp. (Bat Spain) | 0.088 | 0.053 | 0.050 | 0.050 | 0.050 | 0.057 | 0.040 | 0.092 | 0.036 | 0.023 | 0.036 | 0.043 | 0.043 | 0.043 |  |
